# Research Progress on Sesquiterpene Compounds from Artabotrys Plants of Annonaceae

**DOI:** 10.3390/molecules29071648

**Published:** 2024-04-06

**Authors:** Yupei Sun, Jianzeng Xin, Yaxi Xu, Xuyan Wang, Feng Zhao, Changshan Niu, Sheng Liu

**Affiliations:** 1 School of Pharmacy, Yantai University, Yantai 264005, China; syp6935@163.com (Y.S.); unicy81@163.com (Y.X.); 17851072916@163.com (X.W.); 2 School of Life Sciences, Yantai University, Yantai 264005, China; jianzeng77@sina.com; 3 College of Pharmacy, University of Utah, Salt Lake City, UT 84108, USA

**Keywords:** *Artabotrys*, *Annonaceae*, sesquiterpene compounds, biological activities

## Abstract

*Artabotrys*, a pivotal genus within the *Annonaceae* family, is renowned for its extensive biological significance and medicinal potential. The genus’s sesquiterpene compounds have attracted considerable interest from the scientific community due to their structural complexity and diverse biological activities. These compounds exhibit a range of biological activities, including antimalarial, antibacterial, anti-inflammatory analgesic, and anti-tumor properties, positioning them as promising candidates for medical applications. This review aims to summarize the current knowledge on the variety, species, and structural characteristics of sesquiterpene compounds isolated from *Artabotrys* plants. Furthermore, it delves into their pharmacological activities and underlying mechanisms, offering a comprehensive foundation for future research.

## 1. Introduction

*Annonaceae*, a prominent family within the tropical flora, is classified under the *Ranunculaceae*. It contains approximately 130 genera and over 2100 species, featuring a rich diversity of tropical trees, shrubs, and climbing plants [1]. Many species within *Annonaceae* family have been widely used in ethnobotany to treat a myriad of health conditions [2]. For instance, *Polyalthia*, one of the largest and most famous genera within *Annonaceae*, has been widely used in the treatment of rheumatic fever, peptic ulcer, and systemic pain [3]. The chemical diversity present in *Annonaceae* species is vast, yielding a plethora of natural compounds such as alkenes [4], terpenoids [5], alkaloids [6,7,8,9,10], and phenols [11]. These compounds demonstrate a broad spectrum of pharmacological activities, including anti-mosquito [12], anti-cancer [13,14,15], antibacterial [16], anti-protozoal [17,18], and antifungal [19]. Among them, Annonaceous acetogenins stand out for their potent anti-tumor potential, making them one of the most promising natural product discoveries [20].

*Artabotrys*, belonging to the *Annonaceae* family, comprises about 110 species of plants around the world, predominantly distributed in tropical and subtropical regions such as Southeast Asia, Indonesia, and Malaysia. The plants of this genus are climbing shrubs. The leaves of these plants are usually compound, the flowers are small and clustered on the raceme, and the fruits are drupe-shaped. There are many traditional uses of this genus, such as the treatment of cholera, malaria, and other diseases [21]. The plants of this genus have a wide range of biological significance and medicinal value. An examination of data from the Plants of the World Online database facilitated a detailed summary of *Artabotrys* species and their distribution (Table 1).

The genus *Artabotrys*, within the *Annonaceae* family, is distinguished by its wealth of chemical components [22]. To date, research has identified a diverse array of compounds from these plants, including alkaloids [23,24], volatile oils [25,26], cyclohexenes [27,28], phenylpropanoids [29], flavonoids [30], quinones [31], and sesquiterpenes [32]. Among these, sesquiterpenes stand out as one of the principal active components, heralded for their significant medical value and importance in research. So far, there have been many research articles on the plants of *Artabotrys*; however, the majority have focused on individual compounds or relatively extensive research overviews. Comprehensive reviews specifically addressing the sesquiterpene compounds derived from the plants of the genus are notably scarce. Therefore, this paper aims to fill this gap by reviewing the current research progress of sesquiterpene compounds derived from the plants of *Artabotrys* in *Annonaceae*. It meticulously summarizes the variety, species, and structural characteristics of sesquiterpene compounds identified within these plants and explores their pharmacological activities and underlying mechanisms, offering a comprehensive foundation for future research.

## 2. Chemical Constitution

Sesquiterpenes, a diverse class of natural organic compounds, are characterized by a basic carbon skeleton comprising 15 carbon atoms arranged in three isoprene units. Based on the number of carbon rings in the structure, sesquiterpenes can be divided into five structural types: acyclic sesquiterpenes [33], monocyclic sesquiterpenes [34], bicyclic sesquiterpenes [35,36], tricyclic sesquiterpenes [37], and tetracyclic sesquiterpenes [38]. Acyclic sesquiterpenes encompass linear sesquiterpenes [39] and unsaturated acyclic sesquiterpenes [40] whereas monocyclic sesquiterpenes include germacrane [41], cyclofarnesane [42], bisabolane [43], and elemane [44]. Bicyclic sesquiterpenes feature structures like eudesmane [45], isodaucane [46], guaiane [47], acorane [48], and eremophilane [49,50]. Tricyclic sesquiterpenes include aristolane [51], and aromadendrane [52]. Tetracyclic sesquiterpenes include camphane, labdane, and ginkgolide [53,54].

Sesquiterpenes represent a distinguished class of natural organic compounds, notable for their widespread natural sources. These compounds are predominantly derived from a range of plants [55,56], especially those known for their aromatic properties, as well as from fungi [57,58,59], and marine organisms [60,61]. Sesquiterpenes have a variety of biological activities, encompassing antimalarial [62], antioxidant [63], anti-inflammatory [64,65], antibacterial [66,67], and anti-tumor effects [68,69]. Therefore, sesquiterpenes have displayed significant therapeutic potential in the pharmaceutical sector, while their unique properties also make them invaluable to the perfume industry.

Extensive research into the sesquiterpenes extracted from *Artabotrys* plants reveals a remarkable diversity within this genus. To date, investigations have identified over 80 distinct sesquiterpene types isolated from *Artabotrys*, underscoring the genus’s rich contribution to the pool of naturally occurring sesquiterpenes. A detailed breakdown of these sesquiterpenes reveals a wide array of structural types, including 19 bisabolane-type, 15 eudesmane-type, 8 norbisabolane-type, 6 guaiane-type, 4 aromadendrane-type, aristolane-type, and cadinane-type, 3 eremophilane-type, 2 isodaucane-type and acorane-type, 1 germacrane-type, alongside a multitude of other sesquiterpene variants.

These findings further attest to the extraordinary potential of the plant as a ‘natural drug bank’, such as for the development of innovative anti-tumor and anti-inflammatory drugs. Each sesquiterpene identified offers unique insights into potential pharmacological applications and holds the promise of playing a pivotal role in devising novel therapeutic strategies.

### 2.1. Bisabolane-Type Sesquiterpenes

Bisabolane-type sesquiterpenes, a subclass of monocyclic sesquiterpenes, are characterized by their six-membered carbon rings and side chains. These compounds boast a plethora of natural sources, including marine invertebrates, terrestrial plants, and microorganisms. Notably, bisabolane-type sesquiterpenes exhibit a wide range of biological activities, such as anti-inflammatory and antibacterial [70,71]. To date, more than 350 kinds of bisabolane-type sesquiterpenes have been isolated from various plant families, including *Compositae* and *Zingiberaceae* [72]. In the context of the *Artabotrys* genus, several bisabolane-type sesquiterpenes have also been successfully extracted (Table 2).

**Figure 1:**
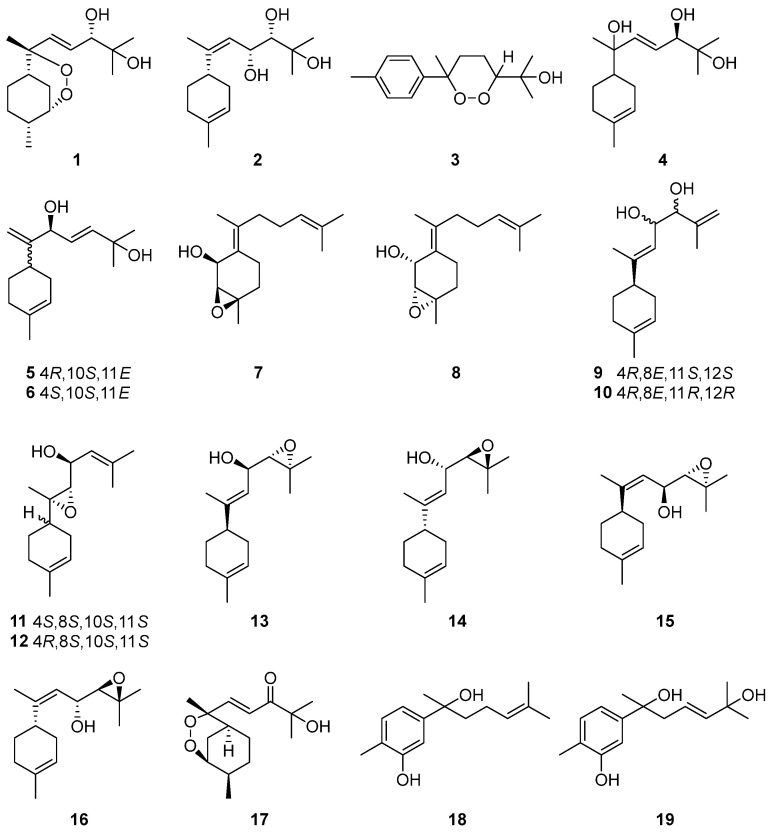
The structures of bisabolane-type sesquiterpenes from *Artabotrys*.

Among the isolated compounds, compounds **1** and **2** were extracted from the roots of *Artabotrys uncinatus* in 1979; their structures were elucidated by spectroscopic methods [73,74]. Compounds **3** and **4** were also derived from *A. uncinatus* [75]. Subsequently, researchers isolated 13 bisabolane-type sesquiterpenes (**5**–**17** in Table 2) from the roots of *A. hexapetalus* in 2017 [76]. Moreover, 25 monomeric compounds, including two bisabolane-type sesquiterpenes chlospicate E (**18**) and arbisabol-9-en-7,11-diol (**19**) [77], were isolated from *Artabotrys pilosus* by a combination of chromatographic separation methods and spectral identification techniques. The structural details of the related compounds are shown in Figure 1.

### 2.2. Norbisabolane-Type Sesquiterpenes

**Figure 2:**
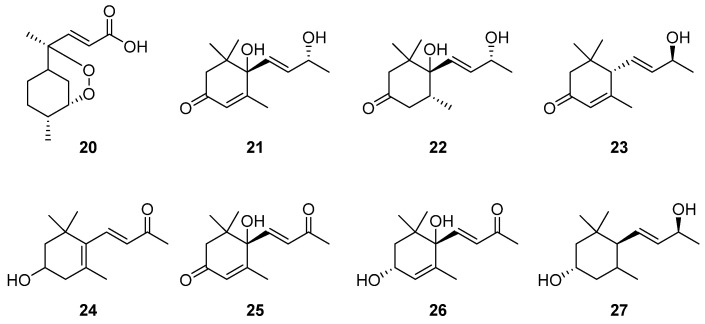
The structures of norbisabolane-type sesquiterpenes from *Artabotrys*.

Norbisabolane-type sesquiterpenes, another subset of monocyclic sesquiterpenes, known for their spiroketal structures, have primarily been isolated from *Phyllanthus* spp. within the *Euphorbiaceae* [78]. From the extracts of *Artabotrys* plants, several norbisabolane-type sesquiterpenes (Table 3) were successfully purified by a series of chromatographic techniques, and the structures were elucidated via comprehensive analysis of nuclear magnetic resonance (NMR), mass spectrometry (MS) and other technical means. Among them, compound **20** was isolated from *A. hexapetalus* [76], while compounds **21**–**27** were isolated from the branches and leaves of *A. hongkongensis* in 2017 [79]. The detailed structures of the compounds are shown in Figure 2.

### 2.3. Eudesmane-Type Sesquiterpenes

Eudesmane-type sesquiterpenes, classified as bicyclic sesquiterpenes, are notable for their widespread distribution in nature. Eudesmane-type sesquiterpenes are characterized by a core structure comprising two six-membered rings and four substituents with a total of 15 carbon atoms, leading to a considerable structural diversity primarily attributed to variations in the substituents’ positioning and the double bonds within the rings. Studies have shown that these compounds displayed anti-inflammatory [80], anti-fungal [81], anti-cancer [82], anti-diabetic nephropathy [83], and the ability to inhibit the proliferation of leukemia cell lines [84].

**Figure 3:**
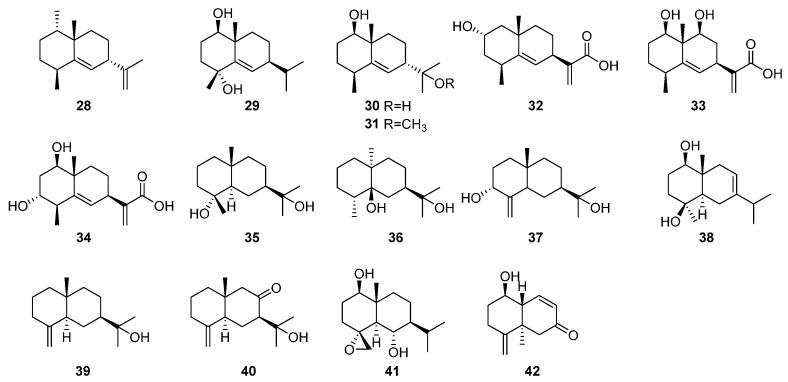
The structures of eudesmane-type sesquiterpenes from *Artabotrys*.

A significant number of eudesmane-type sesquiterpenes have been isolated from *Artabotrys* (Table 4), with compounds **28**–**42** representing this variety. Among them, compounds **28**–**34**, a series of seven eudesmane-type sesquiterpenes, were isolated from *Artabotrys hongkongensis* Hance in 2020 [85]. The compound 7-trinoreudesma-4(15),8-dien-1*β*-ol-7-one (45) was isolated from the ethyl acetate extract of the 90% ethanol extract of the branches and leaves of *A. pilosus* by various modern chromatographic separation techniques. Its identification as colorless oil soluble in chloroform was identified by structural identification, affirming its classification as an eudesmane-type sesquiterpene [77]. Additionally, the other eight eudesmane-type sesquiterpenes (**35**–**42**) were isolated from *Artabotrys hainanensis* [86], *A. hongkongensis* [79], and *A. pilosus* [77] by various separation techniques. The distinctive structures of eudesmane-type sesquiterpenes from the *Artabotrys* genus plants are depicted in Figure 3.

### 2.4. Guaiane-Type Sesquiterpenes

**Figure 4:**
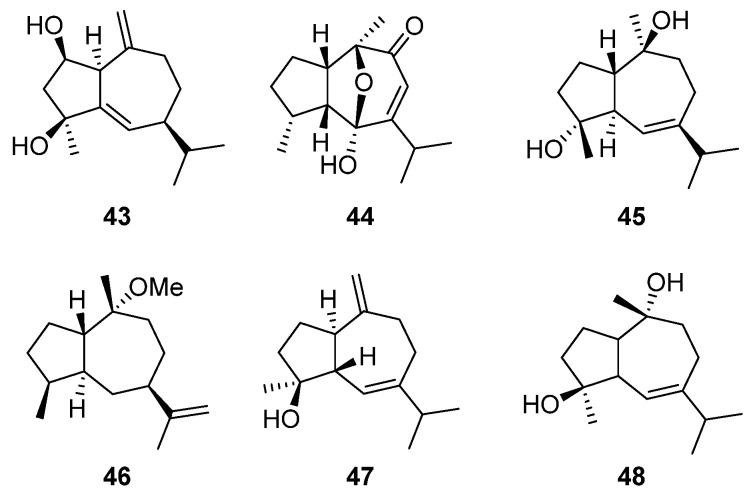
The structures of guaiane-type sesquiterpenes from *Artabotrys*.

Guaiane-type sesquiterpenes, a subclass of bicyclic sesquiterpenes, are distinguished by their unique structural framework, which features a seven-membered ring fused with a five-membered lactone ring, augmented by two methyl groups and one isopropyl group. These compounds are prevalent across more than 30 families of plants, demonstrating a broad spectrum of biological activities, including anti-tumor, anti-inflammatory, antibacterial, and antioxidant [87,88]. The genus *Artabotrys* plants, known for its rich chemical diversity, also harbors guaiane-type sesquiterpenes. Compounds **43**–**48** represent guaiane-type sesquiterpenes isolated from various *Artabotrys* species (Table 5). Guaiane pogostol O-methyl ether (**46**) from *Artabotrys stenopetalus* in 1997 marked the beginning of the identification of such compounds within the genus [89]. Compounds **43** and **44** are two sesquiterpenes isolated from the 90% ethanol extract of the branches and leaves of *A. hainanensis*, both identified as guaiane-type sesquiterpenes [86]. Compound **45**, a colorless oily substance isolated from *A. pilosus* [77], was confirmed as guaianediol through NMR data analysis and comparison with existing literature [90]. Additionally, alismol (**47**) and alismoxide (**48**) were derived from the stem [91] and flower of *A. hainanensis* [86], respectively, with the latter previously identified in *Alisma orientalis* [92]. The structures of these guaiane-type sesquiterpenes are depicted in Figure 4.

### 2.5. Eremophilane-Type Sesquiterpenes

**Figure 5:**
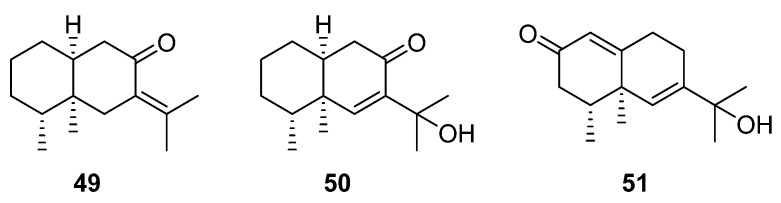
The structures of eremophilane-type sesquiterpenes from *Artabotrys*.

Eremophilane-type sesquiterpenes, derived from the biosynthetic precursor farnesyl diphosphate (FPP), represent a distinct group within the bicyclic sesquiterpene compound family. These compounds are characterized by their unique irregular bicyclic structures, with structural variations primarily arising from various oxidations on the bicyclic skeleton and the isopropyl side chain [93]. Related studies have shown that these compounds displayed anti-inflammatory effects and can inhibit the NO produced by lipopolysaccharide (LPS)-induced RAW 264.7 macrophages [94]. In studying the chemical constituents of the *Artabotrys* genus, researchers have successfully isolated several eremophilane-type sesquiterpenes (Table 6). Among them, compounds **49** and **50** are two eremophilane-type sesquiterpenes obtained from the branches and leaves of *A. hongkongensis* in the same research process [79], while compound **51** was obtained from the branches and leaves of *A. hainanensis* in another study one year later [86]. The chemical structures of these three eremophilane-type sesquiterpenes with serial numbers **49**–**51** are shown in Figure 5.

### 2.6. Isodaucane-Type Sesquiterpenes

**Figure 6:**
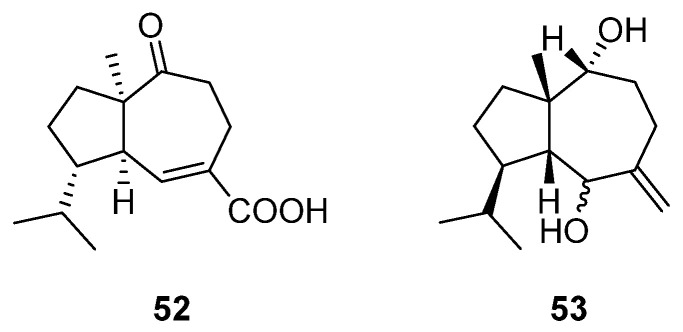
The structures of isodaucane-type sesquiterpenes from *Artabotrys*.

Isodaucane-type sesquiterpenes, which belong to bicyclic sesquiterpenes, are distinguished by their distinctive structural configuration, featuring a five-membered ring coupled with a seven-membered ring. Despite their relatively scarce occurrence in nature compared with other common types of sesquiterpenes, dedicated research efforts have led to the successful isolation of two isodaucane-type sesquiterpenes from the *Artabotrys* genus (Table 7). Compounds **52** and **53** were isolated from the branches and leaves of *A. hongkongensis* and the stem bark of *A. stenopetalus*, respectively [79,89]. The structures of the two compounds are shown in Figure 6.

### 2.7. Acorane-Type Sesquiterpenes

**Figure 7:**
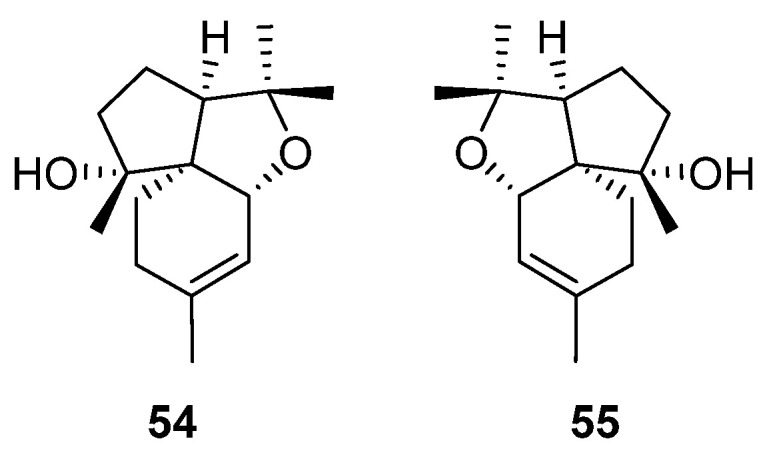
The structures of acorane-type sesquiterpenes from *Artabotrys*.

Acorane-type sesquiterpenes are distinguished by their spiro [4.5] decane skeleton, featuring an isopropyl unit at C-1 and a dimethyl substitution at C-4 and C-8 [95]. This unique natural product category falls within the bicyclic sesquiterpene compound, known for its wide range of pharmacological activities, such as antiviral activity [96] and anti-inflammatory activity [97,98]. Despite their notable bioactivity, acorane-type sesquiterpenes are exceedingly rare in both plants and microorganisms. In a significant discovery, two acorane-type sesquiterpenes were successfully isolated from the genus of *Artabotrys* (Table 8). Compounds **54** and **55** were isolated from the roots of *A. hexapetalus* in 2017 alongside 13 bisabolane-type sesquiterpenes (**5**–**17** in Table 2) [76]. The structures of the two compounds are shown in Figure 7.

### 2.8. Cadinane-Type Sesquiterpenes

**Figure 8:**
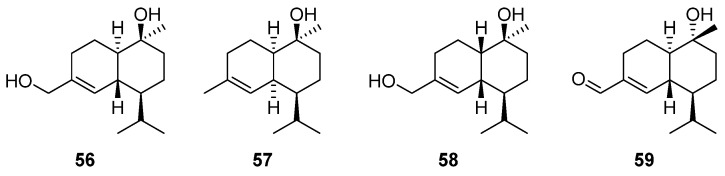
The structures of cadinane-type sesquiterpenes from *Artabotrys*.

Cadinane-type sesquiterpenes, a class of bicyclic sesquiterpenes, are synthesized through the catalytic action of sesquiterpene synthase (STS) on FPP [99]. These compounds have complex stereochemistry and a wide range of pharmacological activities, such as hypoglycemic [100], antifungal [101], and anti-inflammatory [102]. To date, a considerable diversity of cadinane-type sesquiterpenes with diverse structures and biological activities have been isolated and identified from a variety of plants and microorganisms. Furthermore, with the continuous advancement of modern biotechnology, the biosynthetic pathways of representative cadinene-type sesquiterpenes have been substantially elucidated [103]. The following compounds are cadinene-type sesquiterpenes obtained from the genus of *Artabotrys* (Table 9). Notably, 10*β*, 15-hydroxy-*α*-cadinol (**56**) was isolated from both *A. pilosus* [77] and *A. hainanensis* [86]. Additionally, amorph-4-en-10*α*-ol (**57**) was isolated from the branches and leaves of *A. hainanensis* [86]. Compounds **58** and **59**, further enriching the variety of cadinene-type sesquiterpenes, were derived from the branches and leaves of *A. pilosus* [77]. The detailed structures of these compounds are depicted in Figure 8.

### 2.9. Aristolane-Type Sesquiterpenes

**Figure 9:**
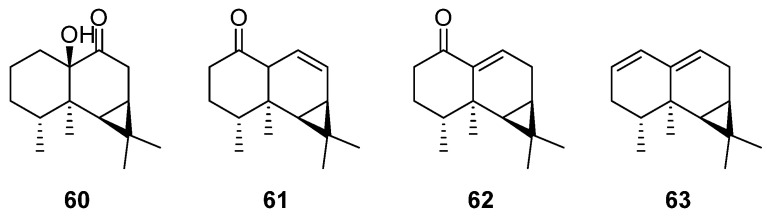
The structures of aristolane-type sesquiterpenes from *Artabotrys*.

Aristolane-type sesquiterpenes are naturally occurring sesquiterpenes, primarily obtained from *Nardostachys*, *Axinyssa*, and *Russula* [104]. Aristolane-type sesquiterpenes usually contain a gem-dimethyl cyclopropane structure [105], which belongs to the tricyclic sesquiterpenes. These compounds play a pivotal role in regulating serotonin transporter (SERT) to enhance or inhibit SERT [106], which offers therapeutic potential for the treatment of neuropsychiatric and digestive diseases. Advances in research and technology have enabled the isolation of several aristolane-type sesquiterpenes from *Artabotrys* plants (Table 10). 10-hydroxyaristolan-9-one (**60**), initially isolated from the stems of *A. uncinatus* in 2007, has also been found in the branches and leaves of *A. hongkongensis* in another study a few years later [79,107], alongside compounds **61**–**63** [79]. The structures of aristolane-type sesquiterpenes involved are shown in Figure 9.

### 2.10. Aromadendrane-Type Sesquiterpenes

**Figure 10:**
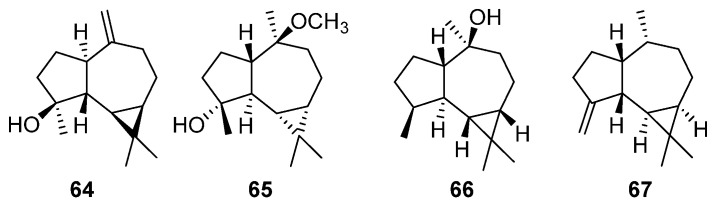
The structures of aromadendrane-type sesquiterpenes from *Artabotrys*.

Aromadendrane-type sesquiterpenes, akin to the aristolane-type sesquiterpenes mentioned earlier, belong to the tricyclic sesquiterpenes family, noted for their anti-inflammatory [108]. Studies have found that certain aromadendrane-type sesquiterpenes compounds can interact with benzoquinone to form heterodimers, offering cytoprotective effects on glutamate-induced neurological deficits [109]. The following three compounds (**64**–**65**) are classified as aromadendrane-type sesquiterpenes obtained from *Artabotrys* (Table 11). Compound **64** was obtained from branches and leaves of *A. hainanensis* [86]. The remaining compound (-)-ent-4*β*-hydroxy-10*α*-methoxyaromadendrane (**65**) was obtained from the stem of *A. uncinatus* by numerous efforts of researchers in 2007 [107]. Compounds **66** and **67** are two sesquiterpenes obtained from the flowers of *A. hexapetalus* [110]. Figure 10 shows the detailed structures of the five aromadendrane-type sesquiterpenes.

### 2.11. Other Types of Sesquiterpenes

Beyond the previously mentioned sesquiterpenes, many other types of sesquiterpenes have also been obtained from the plants of *Artabotrys*, as detailed in Table 12.

**Figure 11:**
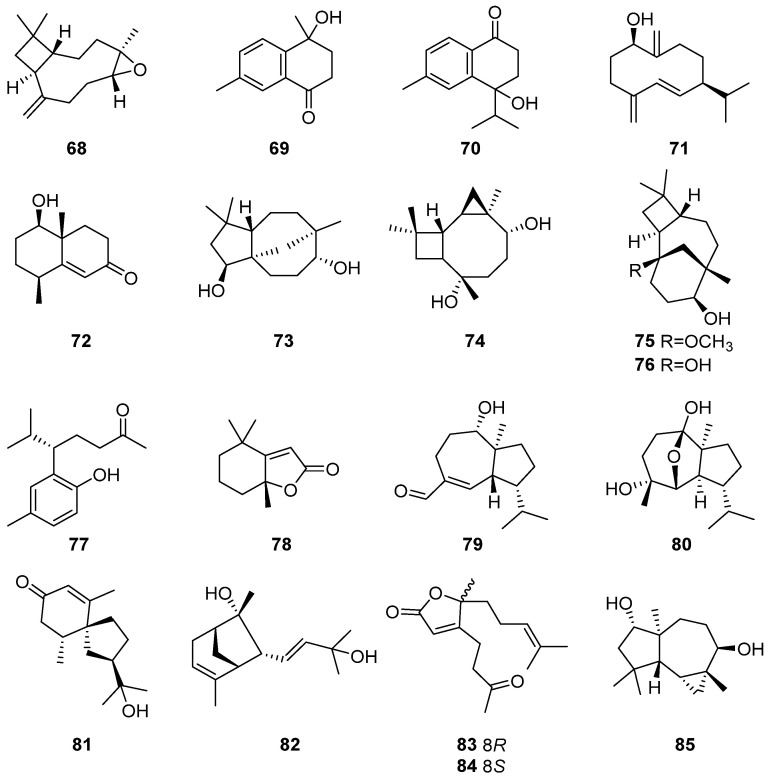
The structures of other types of sesquiterpenes from *Artabotrys*.

Notably, *β*-caryophyllene oxide (**68**), caryophyllene-type sesquiterpenes with a unique polycyclic structure, were isolated from the stem bark of *A. stenopetalus* [89]. Compounds **74** and **76** in Table 12 also belong to this class of sesquiterpenes. Compounds **69** and **70**, derived by reducing some carbon atoms in cadinane-type sesquiterpenes, represent a class of bicyclic sesquiterpene. 4-hydroxy-4,7-dimethyl-1-tetralone **(69**), reduced by 3 carbons, and oxyphyllone D (**70**), reduced by 1 carbon, have been isolated from the branches and leaves of *A. pilosus* [77] and *A. hainanensis* [86], respectively. Additionally, 1*β*-hydroxy-4(15),5*E*,10(14)-germacratriene (**71**), a germacrane-type sesquiterpene, was isolated from the branches and leaves of *A. hainanensis* [86] and belongs to monocyclic sesquiterpenes. artahongkongol A (**72**), a unique trinoreudesmane sesquiterpene derived from the corresponding eudesmane-type sesquiterpenes by removing a propyl group, was obtained from the stems and leaves of *A. hongkongensis* [85]. (4*R*,5*R*,7*R*)-1(10)-spirovetiven-11-ol-2-one (**81**), a rare natural spirovetivane-type sesquiterpene, was first isolated from the flower of *A. hainanensis* [86]. Compounds **82**, **83**, and **84** are three bisabolene-type sesquiterpenes isolated from the roots of *A*. *hexapetalus*, with compounds **83** and **84** identified as a pair of enantiomers [32]. The remaining compounds listed in Table 12, not described in detail here, represent unique sesquiterpenes with special structural types rare in nature isolated from *Artabotrys* plants. The specific structures of the related compounds are illustrated as follows (Figure 11).

## 3. Pharmacological Activities

Sesquiterpenes, with their distinct carbon skeletons and roles in diverse biochemical processes, play a pivotal role in drug discovery and development. Their unique structures enable a wide array of biological and pharmacological actions, making them invaluable in modern medicinal research. In particular, sesquiterpenes from the plants of the genus *Artabotrys* have shown significant activity across numerous pharmacological studies. The following are some key pharmacological activities attributed to sesquiterpenes isolated from *Artabotrys* plants.

### 3.1. Antimalarial Activity

Malaria is one of the oldest diseases in humans. It is a disease caused by parasites [111] mainly transmitted to humans through mosquito bites [112]. Malaria is an infectious disease caused by malaria parasites [113,114,115]. Predominantly prevalent in tropical and subtropical regions, especially in Africa, South Asia, Southeast Asia, and Central America [116], malaria accounts for more than 200 million cases worldwide each year [117]. The development of effective antimalarial drugs can reduce the spread and infection of malaria and accelerate the early recovery of patients [118]. Therefore, it is of great significance to find more effective antimalarial drugs. In the process of studying antimalarial drugs, researchers have found that some sesquiterpenes and some other natural components derived from *Artabotrys* plants have shown antimalarial activity. Notably, yingzhaosu A (**1**) is the first antimalarial drug with a clear structure containing an endoperoxide structure in history [119]. This discovery has spurred further research and the synthesis of new antimalarial drugs, although the exact mechanism of yingzhaosu A’s antimalarial action remains partially understood. Current research suggests that yingzhaosu A’s mechanism of action may involve two primary processes. Firstly, in the presence of oxygen and iron (II), yingzhaosu A will undergo a degradation reaction due to the induction of iron (II), forming unsaturated ketones and cyclohexyl radicals, respectively. The active substances produced in this process may be the reason for its antimalarial effect [120].

Secondly, a recent study found that when yingzhaosu A plays a role in the body, it is attacked by heme, which destroys its peroxide structure, produces tertiary oxygen-centered radicals, and rearranges to remove the side chain. Therefore, the yingzhaosu A is split into two parts. Heme is an important marker of malaria parasites. Based on the above findings, a heme-activatable probe has been successfully developed, which will play an important role in the field of antimalarial [121].

Beyond yingzhaosu A (**1**), related compounds such as yingzhaosu B (**2**), yingzhaosu C (**3**), and yingzhaosu C (**4**) have also demonstrated antimalarial effect, expanding the library of potential antimalarial agents derived from natural sources [122,123,124].

### 3.2. Antibacterial and Antifungal Activity

Bacterial infections significantly impact global health, causing widespread morbidity and mortality, and placing a significant burden on health care systems [125,126]. At present, many bacteria are resistant to antibiotics, which has become an extremely important public health problem [127,128,129]. However, due to the increase in global antimicrobial resistance, the efficacy of some treatments for bacterial infections is reduced or even ineffective. Therefore, it is particularly important to find new therapeutic drugs and design new treatment strategies in the field of antibacterial [130,131].

Among the promising candidates, sesquiterpenes derived from *Artabotrys* plants have demonstrated antibacterial effects through different mechanisms, showing potential against a variety of bacterial and fungal pathogens. Notably, isodaucane-type sesquiterpene artabotrol (**53**), isolated from the stem bark of *A. stenopetalus*, a plant belonging to the genus *Artabotrys*, exhibits a specific inhibitory effect on *Cryptococcus neoformans* [132].

Furthermore, globulol (**66**), isolated from the flowers of *A. hexapetalus* and the fruit of *Eucalyptus globulus* Labill, has been shown to inhibit several fungi, including *Alternaria solani*, *Fusarium oxysporum*, *Fusarium graminearum*, *Rhizoctonia solani*, and *Venturia pirina*, with a half maximal inhibitory concentration (IC_50_) values of 47.1 μM, 114.3 μM, 53.4 μM, 56.9 μM, 32.1 μM, and 21.8 μM, respectively. In addition, the 3-(4,5-dimethylthiazol-2-yl)-2,5-diphenyltetrazolium bromide (MTT) assay results showed that globulol (**66**) also had inhibitory effects on *Xanthomonas vesicatoria* and *Bacillus subtilis*, with IC_50_ values of 158.0 μM and 737.2 μM, respectively [133]. Another compound, dihydroactinidiolide (**78**) showed antibacterial activity against *Bacillus cereus* and *Vibrio parahaemolyticus* in related studies [134].

### 3.3. Antitumor Activity

Some sesquiterpenoids have been shown to have antitumor activity [135]. A notable example is the sesquiterpene (−)-8*R*-Artaboterpenoids B (**83**) isolated from the root of *A. hexapetalus*, which exhibited cytotoxicity against five tumor cells including HCT-116, Hep G2, A2780, NCI-H1650, and BGC-823 with IC_50_ values of 1.38, 3.30, 6.51, 8.19 and 2.14 μM, indicating its potential as an anticancer agent [32]. Similarly, another study identified seven sesquiterpenoids, chlospicate E (**18**), 1*β*, 6*α*-dihydroxy-4*α* (15)-epoxyeudesmane (**41**), guaianediol (**45**), 10*β*,15-hydroxy-*α*-cadinol (**56**), 15-hydroxy-t-muurolol (**58**), 10*α*-hydroxycadin-4-en-15-al (**59**), and 10*β*-hydroxyisodauc-6-en-14-al (**79**) from *A. pilosus* showed significant inhibitory activity against HL-60, SMMC-7721, A-549, MCF-7, and SW480 human tumor cells. These compounds have the potential to develop new anti-tumor drugs as lead compounds. According to the relevant experimental results, the IC_50_ values of chlospicate E (**18**) were 14.25, 21.32, 25.34, 16.23, 10.21 μM, the IC_50_ values of 1*β*, 6*α*-dihydroxy-4*α* (15)-epoxyeudesmane (**41**) were 18.25, 9.65, 8.27, 4.63, 8.64 μM, the IC_50_ values of guaianediol (**45**) were 10.23, 8.64, 9.23, 10.42, 15.22 μM, the IC_50_ values of 10*β*,15-hydroxy-*α*-cadinol (**56**) were 10.11, 5.14, 4.38, 6.32, 3.28 μM, the IC_50_ values of 15-hydroxy-t-muurolol (**58**) were 2.36, 4.02, 7.32, 6.41, 5.23 μM, the IC_50_ values of 10*α*-hydroxycadin-4-en-15-al (**59**) were 5.23, 6.87, 4.96, 5.86, 4.20 μM and the IC_50_ values of 10*β*-hydroxyisodauc-6-en-14-al (**79**) were 15.23, 6.26, 10.23, 9.32, 5.49 μM, respectively. Among these compounds, 10*β*, 15-hydroxy-*α*-cadinol (**56**) had the strongest inhibitory effect on SW480 cells with an IC_50_ value of 3.28 μM, and 1*β*, 6*α*-dihydroxy-4*α* (15)-epoxyeudesmane (**41**) had the strongest inhibitory effect on MCF-7 cells with an IC_50_ value of 4.63 μM [77].

Further research in 2018 unveiled seven eudesmane-type sesquiterpenes (**28**–**34**) and one trinoreudesmane-type sesquiterpene (**72**) from the genus *Artabotrys*, showing cytotoxicity and inhibitory effects on five human tumor cell lines (IC_50_ values of 0.57 to 15.68 μM), with some compounds outperforming the antitumor drug doxorubicin [85]. In addition, yingzhaosu C (**3**) also demonstrated tumor inhibitory effects on HCT-116, HepG 2, and A 2780 cell lines, with IC_50_ values of 3.24, 3.23, and 3.14 μM, respectively [76]. In related studies, compound **24** was found to have a general inhibitory effect on A-549, MCF-7, HT-29, A-498, Pc-3, and PACA-2 human tumor cells, but its effect was not significant, and its IC_50_ values were 11.3, 12.3, 14.5, 16.6, 24.3, and 19.6 μM, respectively [136].

Dihydroactinidiolide (**78**) also has significant anti-tumor activity against four human tumor cell lines, epithelial cell carcinoma (Hela), human prostate cancer (PC-3), breast cancer (MCF-7), and hepatocellular carcinoma (HePG-2) [137]. Additionally, β-caryophyllene oxide (**68**) has been studied for its antitumor mechanism. It is well known that phosphoinositide 3-kinase (PI3K)/protein kinase B (AKT)/mammalian target of rapamycin (mTOR)/ribosomal protein S6 kinase 1 (S6K1) and mitogen-activated protein kinase (MAPK) signaling cascades play an important role in many physiological processes of tumor cells, including cell proliferation, survival, angiogenesis, and metastasis of tumor cells. Through Western blot analysis, MTT assay, and other research methods, it was found that *β*-caryophyllene oxide (**68**) not only inhibited the constitutive activation of PI3K/AKT/mTOR/S6K1 signaling cascade in human prostate cancer PC-3 and breast cancer MCF-7 cells; it also causes the activation of extracellular signal-regulated kinase (ERK), c-Jun N-terminal kinase (JNK) and p38 MAPK in tumor cells, and down-regulates various gene products related to cell proliferation, anti-apoptosis, and metastasis. In addition, in different tumor cells, *β*-caryophyllene oxide (**68**) can simultaneously target PI3K/AKT/mTOR/S6K1 and MAPK signaling pathways, inhibit the proliferation of related tumor cells and induce the apoptosis of tumor cells by activating caspase-3 and releasing cytochrome c. These results suggest that *β*-caryophyllene oxide (**68)** is a potential candidate drug for the prevention and treatment of cancer [138,139,140].

### 3.4. Anti-Inflammatory and Analgesic Activity

Inflammation is a complex immune response, which is the body’s defense mechanism against injury and infection [141,142]. The five main symptoms of inflammation are pain, fever, redness, swelling, and loss of function. Inflammation can be divided into acute and chronic inflammation [143]. If inflammation is left unchecked, it may lead to autoimmune diseases, neurodegenerative diseases, etc. [144]. At present, there are many effective anti-inflammatory drugs, which are also the most common clinical treatment drugs. However, the commonly used anti-inflammatory drugs will have some side effects during the treatment [145,146]. Therefore, in addition to using traditional non-steroidal anti-inflammatory drugs to treat inflammation, some compounds isolated from natural sources are also considered new options for treating inflammatory diseases [147,148,149,150].

Among these, some natural sesquiterpenes obtained from the *Artabotrys* genus have demonstrated promising anti-inflammatory and analgesic activities. For instance, caryolane-1,9*β*-diol (**76**), which was found in *A.uncinatus* in 2007, exhibits significant anti-inflammatory activity in a dose-dependent manner [107,151]. Similarly, spathulenol (**64**), isolated from the twigs and leaves of *A. hainanensis* and previously found in other species such as *Psidium guineense* Sw. has shown notable inhibitory effect on the related pathological symptoms of the Cg-induced paw edema and pleurisy model in mice established in the experiment [152].

Additionally, alismol (**47**) also has anti-inflammatory effects, reducing the levels of NO and prostaglandin E2 in cells and inhibiting the expression of inducible nitric oxide synthase (iNOS) and cyclooxygenase-2 (COX-2) stimulated by lipopolysaccharide in the body. It also inhibits the messenger RNA (mRNA) and protein expression of pro-inflammatory cytokines including interleukin and tumor necrosis factor α (TNF-α) [153].

### 3.5. Antiviral Activity

The ongoing threat of viral infections, such as influenza virus [154], coronavirus disease 2019 (COVID-19) virus [155], and severe acute respiratory syndrome coronavirus 2 (SARS-CoV-2) virus [156], underscores the importance of effective antiviral therapies in preventing disease spread, mitigating viral damage, and facilitating patient recovery. Antiviral drugs not only help control outbreaks and improve treatment outcomes but also minimize the risk of viral mutations and drug resistance. In this context, sesquiterpenes, a class of compounds derived from natural products, hold significant promise for antiviral drug development. Their potential for inhibiting viral activity, supporting drug development, and boosting immunity offers valuable insights for future antiviral strategies.

Research indicates that sesquiterpene compounds **3**, **11**, **12**, **17**, **54**, and **55** have inhibitory effects on Coxsackievirus B3 and influenza A virus. Specifically, compounds **3**, **54**, and **55** have moderate antiviral activity against Coxsackievirus B3, with IC_50_ values ranging from 6.41 to 33.33 μM. Meanwhile, compounds **12** and **17** showed weak inhibitory activity against the influenza A virus with IC_50_ values ranging from 19.24 to 33.33 μM [76]. Furthermore, guaianediol (**45**) obtained from *A. pilosus* in 2016 displayed anti-human immunodeficiency virus type 1 (anti-HIV-1) virus activity. In previous related studies, a variety of research methods have been used to explore its anti-HIV-1 activity, such as human immunodeficiency virus type 1 reverse transcriptase (HIV-1 RT) assay, syncytium assay, and other research methods. In addition to its significant anti-HIV-1 activity in syncytium assay, the results suggest that Guaianediol may inhibit HIV-1 RT, though its exact IC_50_ value requires further investigation [157].

### 3.6. Antioxidant Activity

Studies have shown that some sesquiterpene compounds have significant antioxidant activity [158,159]. These compounds can exhibit antioxidant properties in vivo through a variety of mechanisms, including scavenging free radicals, increasing antioxidant enzyme activity, and regulating oxidation-reduction balance. Among these, spathulenol (**64**) not only exhibits an anti-inflammatory effect but also demonstrates a significant antioxidant effect, with its IC_50_ value ranging from 26.13 to 85.60 μM [152]. Furthermore, studies on the dichloromethane extract of dihydroactinidiolide (**78**) have revealed its free radical scavenging activity, underscoring the antioxidant potential of sesquiterpenes [137].

### 3.7. Discussion on Structure-Activity Relationships

In general, compounds with the same skeleton structure are often possessed of similar biological activities and pharmacological effects. Through comparison and analysis of some compounds with the same skeleton structure, as well as known activities, the possible structure-activity relationship of some sesquiterpene compounds with the same skeleton structure is discussed.

Compounds **56**, **58**, and **59** are cadinane-type sesquiterpenes, with the same skeleton structure. They are all possessed of anti-tumor activities, but the inhibitory effect on the same tumor cells is different. The difference in the structure of compounds **56** and **58** structure is only the difference in the hydrogen atom configuration at the C-1 position. The hydrogen atom at the C-1 position of compound **56** is the *R* configuration, and the hydrogen atom at the C-1 position of **58** is the S configuration. It is speculated that it may be the main factor affecting the pharmacological activity of the two. When the hydrogen atom at the C-1 position of the two is the *R* configuration, this may have a better inhibitory effect on the tumor cells of A-549, MCF-7, and SW480.

## 4. Conclusions

*Artabotrys*, a prominent genus within the *Annonaceae* family, is renowned for its vast global presence and rich chemical diversity, including flavonoids, alkaloids, and terpenoids. Many of the chemical components have shown good pharmacological activity and have high research value.

This paper presents a comprehensive review of the sesquiterpene compounds identified in plants and their pharmacological activities, aiming to provide a solid scientific foundation for further exploring and utilizing this genus. It also seeks to deepen the understanding of sesquiterpene compounds’ pharmacological actions and mechanisms. An extensive review of research literature has cataloged approximately 85 sesquiterpene compounds and their sources from *Artabotrys* plants, categorizing them according to their structural characteristics. In addition to the common types of sesquiterpenes, such as bisabolane-type sesquiterpenes and eudesmane-type sesquiterpenes, which are rich in plant and microbial sources, this genus also harbors sesquiterpenes with special structures that are relatively rare. Pharmacological research reveals that these compounds exhibit a broad spectrum of activities, including antimalarial, anti-inflammatory, antiviral, and antitumor effects, underscoring their significant medicinal potential and positioning them as potential leads for drug development.

Despite the promising pharmacological activity, the mechanism behind the activities of sesquiterpenes from *Artabotrys* plants remains insufficiently explored, posing challenges to their clinical application of the related sesquiterpenes. Furthermore, many sesquiterpene components in the genus of *Artabotrys* remain undiscovered, suggesting vast opportunities for future research. It is anticipated that ongoing studies will uncover new sesquiterpene compounds and elucidate their mechanisms of action, enhancing the therapeutic value of *Artabotrys* sesquiterpenes.

## Figures and Tables

**Table 1:** Distribution of *Artabotrys* plant resources.

No.	Species	Distribution
**1**	*Artabotrys aereus* Ast	Vietnam
**2**	*Artabotrys antunesii* Engl. & Diels	Angola
**3**	*Artabotrys arachnoides* J.Sinclair	New Guinea
**4**	*Artabotrys atractocarpus* I.M.Turner	Borneo
**5**	*Artabotrys aurantiacus* Engl.	Cameroon, Central African Repu, Congo, Gabon, Zaïre
**6**	*Artabotrys blumei* Hook.f. & Thomson	China South-Central, China Southeast, Hainan, Vietnam
**7**	*Artabotrys brachypetalus* Benth.	Botswana, Caprivi Strip, Malawi, Mozambique, Northern Provinces, Tanzania, Zambia, Zaïre, Zimbabwe
**8**	*Artabotrys brevipes* Craib	Laos, Thailand
**9**	*Artabotrys burmanicus* A.DC.	Assam, Myanmar
**10**	*Artabotrys byrsophyllus* I.M.Turner & Utteridge	Malaya, Thailand
**11**	*Artabotrys cagayanensis* Merr.	Philippines
**12**	*Artabotrys camptopetalus* Diels	New Guinea
**13**	*Artabotrys carnosipetalus* Jessup	Queensland
**14**	*Artabotrys caudatus* Wall. ex Hook.f. & Thomson	Assam, Bangladesh, East Himalaya
**15**	*Artabotrys chitkokoi* K.Z.Hein, Naive & J.Chen	Myanmar
**16**	*Artabotrys coccineus* Keay	Nigeria
**17**	*Artabotrys collinus* Hutch.	Tanzania, Zambia
**18**	*Artabotrys congolensis* De Wild. & T.Durand	Cameroon, Central African Repu, Congo, Gabon, Zaïre
**19**	*Artabotrys costatus* King	Borneo, Malaya
**20**	*Artabotrys crassifolius* Hook.f. & Thomson	Malaya, Myanmar, Thailand
**21**	*Artabotrys crassipetalus* Pellegr.	Gabon
**22**	*Artabotrys cumingianus* S.Vidal	Philippines
**23**	*Artabotrys darainensis* Deroin & L.Gaut.	Madagascar
**24**	*Artabotrys dielsianus* Le Thomas	Cameroon
**25**	*Artabotrys fragrans* Jovet-Ast	China South-Central, China Southeast, Vietnam
**26**	*Artabotrys gossweileri* Baker f.	Cabinda
**27**	*Artabotrys gracilis* King	Borneo, Malaya, Sumatera
**28**	*Artabotrys grandifolius* King	Malaya, Sumatera
**29**	*Artabotrys hainanensis* R.E.Fr.	China Southeast, Hainan
**30**	*Artabotrys harmandii* Finet & Gagnep.	Cambodia, Laos, Thailand, Vietnam
**31**	*Artabotrys hexapetalus* (L.f.) Bhandari	Comoros, India, Laos, Sri Lanka
**32**	*Artabotrys hienianus* Bân	Vietnam
**33**	*Artabotrys hildebrandtii* O.Hoffm.	Madagascar
**34**	*Artabotrys hirtipes* Ridl.	Borneo
**35**	*Artabotrys hispidus* Sprague & Hutch.	Guinea, Ivory Coast, Liberia, Sierra Leone
**36**	*Artabotrys inodorus* Zipp.	New Guinea
**37**	*Artabotrys insignis* Engl. & Diels	Benin, Cameroon, Congo, Gabon, Ghana, Guinea, Ivory Coast, Liberia, Sierra Leone, Zaïre
**38**	*Artabotrys insurae* Junhao Chen & Eiadthong	Thailand
**39**	*Artabotrys jacques-felicis* Pellegr.	Cameroon, Central African Repu, Zaïre
**40**	*Artabotrys javanicus* I.M.Turner	Jawa
**41**	*Artabotrys jollyanus* Pierre	Cameroon, Guinea, Ivory Coast, Liberia
**42**	*Artabotrys kinabaluensis* I.M.Turner	Borneo
**43**	*Artabotrys kurzii* Hook.f. & Thomson	Myanmar
**44**	*Artabotrys lanuginosus* Boerl.	Borneo, Sulawesi, Sumatera
**45**	*Artabotrys lastoursvillensis* Pellegr.	Gabon, Uganda
**46**	*Artabotrys letestui* Pellegr.	Congo, Gabon
**47**	*Artabotrys libericus* Diels	Liberia
**48**	*Artabotrys likimensis* De Wild.	Burundi, Central African Repu, Kenya, Rwanda, Uganda, Zaïre
**49**	*Artabotrys longipetalus* Junhao Chen & Eiadthong	Thailand
**50**	*Artabotrys longistigmatus* Nurainas	Sumatera
**51**	*Artabotrys lowianus* King	Malaya
**52**	*Artabotrys luteus* Elmer	Philippines
**53**	*Artabotrys luxurians* Ghesq. ex Cavaco & Keraudr.	Madagascar
**54**	*Artabotrys macrophyllus* Hook.f.	Gulf of Guinea Is.
**55**	*Artabotrys macropodus* I.M.Turner	Borneo
**56**	*Artabotrys madagascariensis* Miq.	Madagascar
**57**	*Artabotrys maingayi* Hook.f. & Thomson	Borneo, Malaya
**58**	*Artabotrys manoranjanii* M.V.Ramana, J.Swamy & K.C.Mohan	Andaman Is.
**59**	*Artabotrys modestus* Diels	Tanzania
**60**	*Artabotrys monteiroae* Oliv.	Angola, Burundi, Ethiopia, Kenya, KwaZulu-Natal, Madagascar, Malawi, Mozambique, Northern Provinces, Rwanda, Sudan, Swaziland, Tanzania, Uganda, Zambia, Zaïre, Zimbabwe
**61**	*Artabotrys multiflorus* C.E.C.Fisch.	China South-Central, China Southeast, Myanmar, Thailand
**62**	*Artabotrys nicobarianus* D.Das	Andaman Is., Nicobar Is.
**63**	*Artabotrys oblanceolatus* Craib	Thailand
**64**	*Artabotrys oblongus* King	Cambodia, Malaya
**65**	*Artabotrys ochropetalus* I.M.Turner	Borneo
**66**	*Artabotrys oliganthus* Engl. & Diels	Cameroon, Central African Repu, Gabon, Guinea, Ivory Coast, Liberia
**67**	*Artabotrys oxycarpus* King	Malaya, Thailand
**68**	*Artabotrys pachypetalus* B.Xue & Junhao Chen	China Southeast
**69**	*Artabotrys pallens* Ast	Vietnam
**70**	*Artabotrys palustris* Louis ex Boutique	Zaïre
**71**	*Artabotrys pandanicarpus* I.M.Turner	Borneo
**72**	*Artabotrys parkinsonii* Chatterjee	Myanmar
**73**	*Artabotrys petelotii* Merr.	Laos, Vietnam
**74**	*Artabotrys phuongianus* Bân	Vietnam
**75**	*Artabotrys pierreanus* Engl. & Diels	Cameroon, Congo, Gabon, Zaïre
**76**	*Artabotrys pilosus* Merr. & Chun	China Southeast, Hainan
**77**	*Artabotrys pleurocarpus* Maingay ex Hook.f. & Thomson	Malaya, Thailand
**78**	*Artabotrys polygynus* Miq.	Borneo
**79**	*Artabotrys porphyrifolius* Nurainas	Sumatera
**80**	*Artabotrys punctulatus* C.Y.Wu	China South-Central, Thailand
**81**	*Artabotrys rhynchocarpus* C.Y.Wu	China South-Central, China Southeast
**82**	*Artabotrys roseus* Boerl.	Borneo
**83**	*Artabotrys rufus* De Wild.	Benin, Cameroon, Central African Repu, Congo, Gabon, Nigeria, Togo, Zaïre
**84**	*Artabotrys rupestris* Diels	Tanzania
**85**	*Artabotrys sahyadricus* Robi, K.M.P.Kumar & Hareesh	India
**86**	*Artabotrys sarawakensis* I.M.Turner	Borneo
**87**	*Artabotrys scortechinii* King	Malaya
**88**	*Artabotrys scytophyllus* (Diels) Cavaco & Keraudren	Madagascar
**89**	*Artabotrys sericeus* Sujana & Vadhyar	India
**90**	*Artabotrys siamensis* Miq.	Myanmar, Thailand
**91**	*Artabotrys spathulatus* Jun H.Chen, Chalermglin & R.M.K.Saunders	Thailand
**92**	*Artabotrys speciosus* Kurz ex Hook.f. & Thomson	Andaman Is.
**93**	*Artabotrys spinosus* Craib	Cambodia, Laos, Thailand, Vietnam
**94**	*Artabotrys suaveolens* (Blume) Blume	Borneo, Jawa, Lesser Sunda Is., Malaya, Maluku, Myanmar, New Guinea, Nicobar Is., Philippines, Sulawesi, Sumatera, Thailand, Bangladesh
**95**	*Artabotrys sumatranus* Miq.	Borneo, Jawa, Sumatera
**96**	*Artabotrys tanaosriensis* Jun H.Chen, Chalermglin & R.M.K.Saunders	Thailand
**97**	*Artabotrys taynguyenensis* Bân	Vietnam
**98**	*Artabotrys tetramerus* Bân	Vietnam
**99**	*Artabotrys thomsonii* Oliv.	Cabinda, Cameroon, Central African Repu, Congo, Gabon, Liberia, Nigeria, Zaïre
**100**	*Artabotrys tipulifer* I.M.Turner & Utteridge	Malaya, Thailand
**101**	*Artabotrys tomentosus* Nurainas	Sumatera
**102**	*Artabotrys uniflorus* (Griff.) Craib	Myanmar, Thailand
**103**	*Artabotrys veldkampii* I.M.Turner	Borneo
**104**	*Artabotrys velutinus* Scott Elliot	Benin, Cabinda, Cameroon, Central African Repu, Congo, Gabon, Ghana, Guinea, Guinea-Bissau, Ivory Coast, Liberia, Nigeria, Senegal, Sierra Leone, Uganda, Zaïre
**105**	*Artabotrys venustus* King	Borneo, Malaya, Sumatera, Thailand
**106**	*Artabotrys vidalianus* Elmer	Philippines
**107**	*Artabotrys vietnamensis* Bân	Vietnam
**108**	*Artabotrys vinhensis* Ast	Vietnam
**109**	*Artabotrys wrayi* King	Malaya
**110**	*Artabotrys zeylanicus* Hook.f. & Thomson	India, Sri Lanka

**Table 2:** Bisabolane-type sesquiterpenes from *Artabotrys*.

No.	Name of Compound	Source	Reference
**1**	Yingzhaosu A	*A. uncinatus*	[73]
**2**	Yingzhaosu B	*A. uncinatus*	[74]
**3**	Yingzhaosu C	*A. uncinatus*	[75]
**4**	Yingzhaosu D	*A. uncinatus*	[75]
**5**	(4*R*,10*S*,11*E*)-Yingzhaosu F	*A. hexapetalus*	[76]
**6**	(4*S*,10*S*,11*E*)-Yingzhaosu F	*A. hexapetalus*	[76]
**7**	(*1R*,2*S*,3*S*,4*E*)-Yingzhaosu G	*A. hexapetalus*	[76]
**8**	(*1S*,2*R*,3*R*,4*E*)-Yingzhaosu G	*A. hexapetalus*	[76]
**9**	(4*R*,8*E*,11*S*,12*S*)-Yingzhaosu H	*A. hexapetalus*	[76]
**10**	(4*R*,8*E*,11*R*,12*R*)-Yingzhaosu H	*A. hexapetalus*	[76]
**11**	(4*S*,*8S*,10*S*,11*S*)-Yingzhaosu I	*A. hexapetalus*	[76]
**12**	(4*R*,8*S*,10*S*,11*S*)-Yingzhaosu I	*A. hexapetalus*	[76]
**13**	(4*R*,8*E*,11*R*,12*S*)-Yingzhaosu J	*A. hexapetalus*	[76]
**14**	(4*S*,8*E*,11*S*,12*R*)-Yingzhaosu J	*A. hexapetalus*	[76]
**15**	(4*R,8Z*,11*S*,12*S*)-Yingzhaosu K	*A. hexapetalus*	[76]
**16**	(*4S*,8*Z*,11*R*,12*R*)-Yingzhaosu K	*A. hexapetalus*	[76]
**17**	(1*S*,2*R*,4*R*,8*S*,10*E*)-Yingzhaosu L	*A. hexapetalus*	[76]
**18**	Chlospicate E	*A. pilosus*	[77]
**19**	Arbisabol-9-en-7,11-diol	*A. pilosus*	[77]

**Table 3:** Norbisabolane-type sesquiterpenes from *Artabotrys*.

No.	Name of Compound	Source	Reference
**20**	(1*R*,2*S*,4*R*,8*R*,10*E*)-Yingzhaosu M	*A. hexapetalus*	[77]
**21**	Blumenol A	*A. hongkongensis*	[79]
**22**	4,5-Dihydroblumenol A	*A. hongkongensis*	[79]
**23**	(6*R*,9*S*)-3-Oxo-*α*-ionol	*A. hongkongensis*	[79]
**24**	3-Hydroxy-*β*-ionone	*A. hongkongensis*	[79]
**25**	Dehydrovomifoliol	*A. hongkongensis*	[79]
**26**	(3*R*,6*R*,7*E*)-3-Hydroxy-4,7-Megastigmadien-9-one	*A. hongkongensis*	[79]
**27**	Sarmentol F	*A. hongkongensis*	[79]

**Table 4:** Eudesmane-type sesquiterpenes from *Artabotrys*.

No.	Name of Compound	Source	Reference
**28**	1*α*-Hydroxy-5,11-eudesmadiene	*A. hongkongensis*	[85]
**29**	5-Eudesmene-1*β*,4*α*-diol	*A. hongkongensis*	[85]
**30**	1*β*,11-Dihydroxy-5-eudesmene	*A. hongkongensis*	[85]
**31**	1*β*-Hydroxy-11-methoxy-5-eudesmene	*A. hongkongensis*	[85]
**32**	2*α*-Hydroxy pterodontic acid	*A. hongkongensis*	[85]
**33**	1*β*,9*β*-Dihydroxy-4aH-eudesma-5,11(13)-Dien-12-oic acid	*A. hongkongensis*	[85]
**34**	1*β*,3*α*-Dihydroxyeudesma-5,11(13)-Dien-12-oic acid	*A. hongkongensis*	[85]
**35**	Cryptomeridiol	*A. hainanensis*	[86]
**36**	4,10-Epi-5*β*-hydroxydihydroeiidesmol	*A. hainanensis*	[86]
**37**	Eudesm-4(14)-ene-3*α*,11-diol	*A. hainanensis*	[86]
**38**	Oplodiol	*A. hainanensis*	[86]
**39**	*β*-Eudesmol	*A. hainanensis* *A. hongkongensis*	[86] [79]
**40**	Trans-3*β*-(1-hydroxy-1-methylethyl)-8*αβ*-methyl-5-methylenedecalin-2-one	*A. hongkongensis*	[79]
**41**	1*β*,6*α*-Dihydroxy-4*α* (15)-Epoxyeudesmane	*A. pilosus*	[77]
**42**	7-Trinoreudesma-4(15),8-dien-1*β*-ol-7-one	*A. pilosus*	[77]

**Table 5:** Guaiane-type sesquiterpenes from *Artabotrys*.

No.	Name of Compound	Source	Reference
**43**	Liguducin A	*A. hainanensis*	[86]
**44**	Alpinenone	*A. hainanensis*	[86]
**45**	Guaianediol	*A. pilosus*	[77]
**46**	Guaiane pogostol *O*-methyl ether	*A. stenopetalus*	[89]
**47**	Alismol	*A. hainanensis*	[91]
**48**	Alismoxide	*A. hainanensis*	[86]

**Table 6:** Eremophilane-type sesquiterpenes from *Artabotrys*.

No.	Name of Compound	Source	Reference
**49**	Fukinone	*A. hongkongensis*	[79]
**50**	Petasitolone	*A. hongkongensis*	[79]
**51**	11-Hydroxy-valenc-1(10)-en-2-one	*A. hainanensis*	[86]

**Table 7:** Isodaucane-type sesquiterpenes from *Artabotrys*.

No.	Name of Compound	Source	Reference
**52**	10-Oxo-isodauc-3-en-15-oic acid	*A. hongkongensis*	[79]
**53**	Artabotrol	*A. stenopetalus*	[89]

**Table 8:** Acorane-type sesquiterpenes from *Artabotrys*.

No.	Name of Compound	Source	Reference
**54**	(3*R*,4*S*,8*R*,12*R*)-Yingzhaosu E	*A. hexapetalus*	[76]
**55**	(3*S*,4*R*,8*S*,12*S*)-Yingzhaosu E	*A. hexapetalus*	[76]

**Table 9:** Cadinane-type sesquiterpenes from *Artabotrys*.

No.	Name of Compound	Source	Reference
**56**	10*β*,15-Hydroxy-*α*-cadinol	*A. pilosus* *A. hainanensis*	[77] [86]
**57**	Amorph-4-en-10*α*-ol	*A. hainanensis*	[86]
**58**	15-Hydroxy-t-muurolol	*A. pilosus*	[77]
**59**	10*α*-Hydroxycadin-4-en-15-al	*A. pilosus*	[77]

**Table 10:** Aristolane-type sesquiterpenes from *Artabotrys*.

No.	Name of Compound	Source	Reference
**60**	10-Hydroxyaristolan-9-one	*A. uncinatus* *A. hongkongensis*	[107] [79]
**61**	Aristol-8-en-1-one	*A. hongkongensis*	[79]
**62**	Aristolan-9-en-1-one	*A. hongkongensis*	[79]
**63**	Aristolan-1,9-diene	*A. hongkongensis*	[79]

**Table 11:** Aromadendrane-type sesquiterpenes from *Artabotrys*.

No.	Name of Compound	Source	Reference
**64**	Spathulenol	*A. hainanensis*	[86]
**65**	(-)-Ent-4*β*-hydroxy-10*α*-Methoxyaromadendrane	*A. uncinatus*	[107]
**66**	Globulol	*A. hexapetalus*	[110]
**67**	*β*-Gurjunene	*A. hexapetalus*	[110]

**Table 12:** Other types of sesquiterpenes from *Artabotrys*.

No.	Name of Compound	Source	Reference
**68**	*β*-Caryophyllene oxide	*A. stenopetalus*	[89]
**69**	4-Hydroxy-4,7-dimethyl-1-tetralone	*A. pilosus* *A. hainanensis*	[77] [86]
**70**	Oxyphyllone D	*A. pilosus*	[77]
**71**	1*β*-Hydroxy-4(15),5*E*,10(14)-germacratriene	*A.hainanensis*	[86]
**72**	Artahongkongol A	*A. hongkongensis*	[85]
**73**	Clovane-2*β*,9*α*-diol	*A. hainanensis*	[91]
**74**	Tricyclohumuladiol	*A. hainanensis*	[91]
**75**	1-Methoxy-9-caryolanol	*A. uncinatus*	[107]
**76**	Caryolane-1,9*β*-diol	*A. uncinatus*	[107]
**77**	Litseachromolaevane A	*A. hainanensis*	[86]
**78**	Dihydroactinidiolide	*A. hainanensis*	[86]
**79**	10*β*-Hydroxyisodauc-6-en-14-al	*A. pilosus* *A. hainanensis*	[77] [86]
**80**	Homalomenol C	*A. hainanensis*	[86]
**81**	(4*R*,5*R*,7*R*)-1(10)-spirovetiven-11-ol-2-one	*A. hainanensis*	[86]
**82**	(2*R*,4*S*,8*S*,10*R*)-Artaboterpenoid A	*A. hexapetalus*	[32]
**83**	(−)-8*R*-Artaboterpenoid B	*A. hexapetalus*	[32]
**84**	(+)-8*S*-Artaboterpenoid B	*A. hexapetalus*	[32]
**85**	Junipediol	*A. hainanensis*	[91]

## Data Availability

No new data were created or analyzed in this study. Data sharing is not applicable to this article.

## Abbreviations

NMR	Nuclear magnetic resonance
FPP	Farnesyl diphosphate
LPS	Lipopolysaccharide
STS	Sesquiterpene synthase
SERT	Regulating serotonin transporter
IC_50_	Half maximal inhibitory concentration
MTT	3-(4,5-dimethylthiazol-2-yl)-2,5-diphenyltetrazolium bromide
PI3K	Phosphoinositide 3-kinase
AKT	Protein kinase B
mTOR	Mammalian target of rapamycin
S6K1	Ribosomal protein S6 kinase 1
MAPK	Mitogen-activated protein kinase
ERK	Extracellular signal-regulated kinase
JNK	c-Jun N-terminal kinase
iNOS	Inducible nitric oxide synthase
COX-2	Cyclooxygenase-2
mRNA	Messenger RNA
TNF-α	Tumor necrosis factor α
COVID-19	Coronavirus disease 2019
SARS-CoV-2	Severe acute respiratory syndrome coronavirus 2
anti-HIV-1	Anti-human immunodeficiency virus type 1
HIV-1 RT	Human immunodeficiency virus type 1 reverse transcriptase

## References

[1] Cascaes M.M., Carneiro O.D., Nascimento L.D., de Moraes Â.A., de Oliveira M.S., Cruz J.N., Guilhon G.M., Andrade E.H. (2021). Essential Oils from *Annonaceae* Species from Brazil: A Systematic Review of Their Phytochemistry, and Biological Activities. Int. J. Mol. Sci..

[2] Jourjine I.A.P., Bauernschmidt C., Müller C., Bracher F. (2022). A GC-MS Protocol for the Identification of Polycyclic Aromatic Alkaloids from *Annonaceae*. Molecules.

[3] Yao L.J., Jalil J., Attiq A., Hui C.C., Zakaria N.A. (2019). The medicinal uses, toxicities and anti-inflammatory activity of *Polyalthia* species (*Annonaceae*). J. Ethnopharmacol..

[4] Santos A.C.d., Nogueira M.L., Oliveira F.P.d., Costa E.V., Bezerra D.P. (2022). Essential Oils of *Duguetia* Species A. St. Hill (*Annonaceae*): Chemical Diversity and Pharmacological Potential. Biomolecules.

[5] Araújo C.d.S., Nery D.A., Oliveira A.P.d., Oliveira-Júnior R.G.d., Rolim L.A., Lopes N.P., Silva M.F.d.S., Pessoa C.d.Ó., Braz-Filho R., Dutra L.M. (2023). New *ent*-kaurene-type nor-diterpene and other compounds isolated from *Annona vepretorum* Mart. (*Annonaceae*). Nat. Prod. Res..

[6] Lorenzo V.P., Scotti L., da Silva Almeida J.R.G., Scotti M.T. (2020). *Annonaceae* Family Alkaloids as Agents against Leishmaniasis: A Review and Molecular Docking Evaluation. Curr. Drug Metab..

[7] Lúcio A.S., Almeida J.R., Da-Cunha E.V., Tavares J.F., Barbosa Filho J.M. (2015). Alkaloids of the *Annonaceae*: Occurrence and a compilation of their biological activities. Alkaloids Chem. Biol..

[8] Lorenzo V.P., Lúcio A.S., Scotti L., Tavares J.F., Filho J.M., Lima T.K., Rocha J.D., Scotti M.T. (2016). Structure-and Ligand-Based Approaches to Evaluate Aporphynic Alkaloids from *Annonaceae* as Multi-Target Agent against Leishmania donovani. Curr. Pharm. Des..

[9] Nguemdjo Chimeze V.W., Bankoglu E.E., Zühlke S., Fannang V.S., Eckelmann D., Chi Shirri J., Djuidje E.N., Djama C.M., Stopper H., Wandji J. (2022). Cytotoxic and genotoxic properties of artathomsonine, a new oxoberberine alkaloid from *Artabotrys thomsonii* (*annonaceae*). Nat. Prod. Res..

[10] Huynh N.V., Nguyen Huu D.M., Huynh N.T., Chau D.H., Nguyen C.D., Nguyen Truong Q.D., Mai D.T., Dang P.H. (2022). Anonazepine, a new alkaloid from the leaves of *Annona muricata* (*Annonaceae*). Z. Naturforschung C J. Biosci..

[11] de Moraes M.R., Ryan S.M., Godoy H.T., Thomas A.L., Maia J.G.S., Richards K.M., Tran K., Smith R.E. (2020). Phenolic Compounds and Metals in Some Edible *Annonaceae* Fruits. Biol. Trace Elem. Res..

[12] Maia D.S., Lopes C.F., Saldanha A.A., Silva N.L., Sartori Â.L.B., Carollo C.A., Sobral M.G., Alves S.N., Silva D.B., de Siqueira J.M. (2020). Larvicidal effect from different *Annonaceae* species on Culex quinquefasciatus. Environ. Sci. Pollut. Res..

[13] Rady I., Bloch M.B., Chamcheu R.N., Banang Mbeumi S., Anwar M.R., Mohamed H., Babatunde A.S., Kuiate J.R., Noubissi F.K., El Sayed K.A. (2018). Anticancer Properties of Graviola (*Annona muricata*): A Comprehensive Mechanistic Review. Oxid. Med. Cell. Longev..

[14] Costa E.V., de Souza C.A.S., Galvão A.F.C., Silva V.R., Santos L.d.S., Dias R.B., Rocha C.A.G., Soares M.B.P., da Silva F.M.A., Koolen H.H.F. (2022). *Duguetia pycnastera* Sandwith (*Annonaceae*) Leaf Essential Oil Inhibits HepG2 Cell Growth In Vitro and In Vivo. Molecules.

[15] Galvão A.F.C., Araújo M.d.S., Silva V.R., Santos L.d.S., Dias R.B., Rocha C.A.G., Soares M.B.P., Silva F.M.A.d., Koolen H.H.F., Zengin G. (2022). Antitumor Effect of *Guatteria olivacea* R. E. Fr. (*Annonaceae*) Leaf Essential Oil in Liver Cancer. Molecules.

[16] Terezan A.P., Junqueira J.G.M., Wakui V.G., Kato L., Oliveira C.M.A., Martins C.H.G., Santiago M.B., Severino V.G.P. (2022). Qualitative analysis of the acetogenins from *Annona coriacea* (*Annonaceae*) leaves by HPLC-Q-Orbitrap and their antibacterial potential against oral pathogens. Nat. Prod. Res..

[17] Aminimoghadamfarouj N., Nematollahi A., Wiart C. (2011). *Annonaceae*: Bio-resource for tomorrow’s drug discovery. J. Asian Nat. Prod. Res..

[18] Okpekon T.A., Kabran F.A., Say V.M., Evanno L., Maciuk A., Loiseau P., Champy P., Figadère B. (2021). Apoprunellelactone (APL), an antiprotozoal lactone from the stem barks of *Isolona cooperi* Hutch. & Dalziel (*Annonaceae*). Nat. Prod. Res..

[19] Kayo M.T., Simo M.K., Tagatsing Fotsing M., Talla E., Laurent S., Elst L.V., Henoumont C., Yankep E., Alfred Ngenge T., Keumoe R. (2021). Antifungal potential of extracts, fractions and compounds from *Uvaria comperei* (*Annonaceae*) and *Oxyanthus unilocularis* (Rubiaceae). Nat. Prod. Res..

[20] Tundis R., Xiao J., Loizzo M.R. (2017). *Annona* species (*Annonaceae*): A rich source of potential antitumor agents?. Ann. N. Y. Acad. Sci..

[21] Andriamadio J.H., Rasoanaivo L.H., Benedec D., Vlase L., Gheldiu A.M., Duma M., Toiu A., Raharisololalao A., Oniga I. (2015). HPLC/MS analysis of polyphenols, antioxidant and antimicrobial activities of *Artabotrys hildebrandtii* O. Hffm. extracts. Nat. Prod. Res..

[22] Quang Hop N., The Son N. (2022). Botanical Description, Traditional Uses, Phytochemistry, and Pharmacology of the Genus *Artabotrys*: A Review. Chem. Biodivers..

[23] Hsieh T.J., Chang F.R., Chia Y.C., Chen C.Y., Lin H.C., Chiu H.F., Wu Y.C. (2001). The Alkaloids of *Artabotrys uncinatus*. J. Nat. Prod..

[24] Tan K.K., Khoo T.J., Rajagopal M., Wiart C. (2015). Antibacterial alkaloids from *Artabotrys crassifolius* Hook.f. & Thomson. Nat. Prod. Res..

[25] Wong H.F., Brown G.D. (2002). β-Methoxy-γ-methylene-α,β-unsaturated-γ-butyrolactones from *Artabotrys hexapetalus*. Phytochemistry.

[26] Murphy B.T., Cao S., Brodie P.J., Miller J.S., Ratovoson F., Birkinshaw C., Rakotobe E., Rasamison V.E., Tendyke K., Suh E.M. (2008). Antiproliferative compounds of *Artabotrys madagascariensis* from the *Madagascar rainforest*. Nat. Prod. Res..

[27] Tang J.Y., Liu Y.P., Ju P.K., Luo X.L., Zhang Z.J., Ren P., Lai L., Chen G.Y., Fu Y.H. (2018). A new polyoxygenated cyclohexene derivative from *Artabotrys hainanensis*. Nat. Prod. Res..

[28] Liu Y.P., Tang J.Y., Hua Y., Lai L., Luo X.L., Zhang Z.J., Yin W.Q., Chen G.Y., Fu Y.H. (2018). Bioactive polyoxygenated seco-cyclohexenes from *Artabotrys hongkongensis*. Bioorg. Chem..

[29] Jingguang Y., Tongmei L., Lan S., Xiuzhen L. (2002). Neo-Lignans and Hemiterpenoid from the Seeds of *Artabostrys hexapetalus* (*Annonaceae*). J. Chin. Pharm. Sci..

[30] Somanawat J., Talangsri N., Deepolngam S., Kaewamatawong R. (2012). Flavonoid and megastigmane glycosides from *Artabotrys hexapetalus* leaves. Biochem. Syst. Ecol..

[31] Sharma M., Singh N., Jafri M., Mehta B. (2005). Anthraquinones from *Artabotrys odoratissimus* (Leaves). Indian J. Chem..

[32] Xi F.M., Ma S.G., Liu Y.B., Li L., Yu S.S. (2016). Artaboterpenoids A and B, Bisabolene-Derived Sesquiterpenoids from *Artabotrys hexapetalus*. Org. Lett..

[33] Achoub H., Mencherini T., Esposito T., Luca R., Aquino R., Gazzerro P., Zaiter L., Benayache F., Benayache S. (2021). New sesquiterpenes from *Asteriscus graveolens*. Nat. Prod. Res..

[34] Eddin L.B., Jha N.K., Goyal S.N., Agrawal Y.O., Subramanya S.B., Bastaki S.M.A., Ojha S. (2022). Health Benefits, Pharmacological Effects, Molecular Mechanisms, and Therapeutic Potential of α-Bisabolol. Nutrients.

[35] Scandiffio R., Geddo F., Cottone E., Querio G., Antoniotti S., Gallo M.P., Maffei M.E., Bovolin P. (2020). Protective Effects of (E)-β-Caryophyllene (BCP) in Chronic Inflammation. Nutrients.

[36] Su W., Zhao J.P., Hu J., Yang M., Jacob M., Cai X., Zeng R., Chen S.H., Huang H.Y., Khan I. (2014). Two new bicyclic sesquiterpenes from the stems of *Kadsura heteroclita*. Nat. Prod. Res..

[37] Le Bideau F., Kousara M., Chen L., Wei L., Dumas F. (2017). Tricyclic Sesquiterpenes from Marine Origin. Chem. Rev..

[38] Hemtasin C., Kanokmedhakul S., Kanokmedhakul K., Hahnvajanawong C., Soytong K., Prabpai S., Kongsaeree P. (2011). Cytotoxic Pentacyclic and Tetracyclic Aromatic Sesquiterpenes from *Phomopsis archeri*. J. Nat. Prod..

[39] Dai Q., Zhang F.L., Feng T. (2021). Sesquiterpenoids Specially Produced by Fungi: Structures, Biological Activities, Chemical and Biosynthesis (2015–2020). J. Fungi.

[40] Durairaj J., Di Girolamo A., Bouwmeester H.J., de Ridder D., Beekwilder J., van Dijk A.D. (2019). An analysis of characterized plant sesquiterpene synthases. Phytochemistry.

[41] Yan H., Xu L.L., Zheng X.F., Zou X.F., Xiao L.G., Zhou Y.S., He L., Liu H.Y. (2024). Sesquiterpenes from Chloranthus holostegius with anti-inflammatory activities. Fitoterapia.

[42] Rajachan O.a., Sangdee A., Kanokmedhakul K., Tontapha S., Amornkitbamrung V., Kanokmedhakul S. (2020). Cyclofarnesane sesquiterpenoids from the fungus *Sanghuangporus* sp.. Phytochem. Lett..

[43] Zou J.X., Song Y.P., Liu X.H., Li X.N., Ji N.Y. (2021). Bisabolane, cadinane, and cyclonerane sesquiterpenes from an algicolous strain of *Trichoderma asperelloides*. Bioorg. Chem..

[44] Ding L.F., Liu J.X., Xie Z.Q., Wang D.S., Nie W., Song L.D., Wu X.D., Zhao Q.S. (2019). Magnograndins J-M, elemane sesquiterpenoids from the leaves of *Magnolia grandiflora* and their inhibitory effects on nitric oxide production. Phytochem. Lett..

[45] Wu Q.X., Shi Y.P., Jia Z.J. (2006). Eudesmane sesquiterpenoids from the *Asteraceae* family. Nat. Prod. Rep..

[46] Tan Y.P., Savchenko A.I., Agnew-Francis K.A., Boyle G.M., Bernhardt P.V., Fraser J.A., Williams C.M. (2020). Kalparinol, a Salvialane (Isodaucane) Sesquiterpenoid Derived from Native Australian *Dysphania* Species That Suggests a Putative Biogenetic Link to Zerumbone. J. Nat. Prod..

[47] Liu J.M., Zhang D.W., Du W.Y., Zhang M., Zhao J.L., Chen R.D., Xie K.B., Dai J.G. (2022). Sesquiterpenes from the endophytic fungus *Periconia* sp. F-31. J. Asian Nat. Prod. Res..

[48] Li J., Zhao J., Wang W., Li L., Zhang L., Zhao X.F., Liu Q.R., Liu F., Yang M., Khan I.A. (2017). New Acorane-Type Sesquiterpene from *Acorus calamus* L.. Molecules.

[49] Yuyama K.T., Fortkamp D., Abraham W.R. (2017). Eremophilane-type sesquiterpenes from fungi and their medicinal potential. Biol. Chem..

[50] Arizmendi N., Hou C., Guo F., Li Y., Kulka M. (2018). Bicyclic eremophilane-type petasite sesquiterpenes potentiate peroxisome proliferator-activated receptor γ activator-mediated inhibition of dendritic cells. Int. J. Immunopathol. Pharmacol..

[51] Wang Q., Tang X., Liu H., Luo X., Sung P.J., Li P., Li G. (2020). Clavukoellians G–K, New Nardosinane and Aristolane Sesquiterpenoids with Angiogenesis Promoting Activity from the Marine Soft Coral *Lemnalia* sp.. Mar. Drugs.

[52] Buechi G., Hofheinz W., Paukstelis J.V. (1969). Synthesis of (-)-aromadendrene and related sesquiterpenes. J. Am. Chem. Soc..

[53] Li C., Liu K., Liu S., Aerqin Q., Wu X. (2020). Role of Ginkgolides in the Inflammatory Immune Response of Neurological Diseases: A Review of Current Literatures. Front. Syst. Neurosci..

[54] Hien N.T., Cuc D.T., Thuy N.T.T., Hiep H., Huyen V.T., Ai D.T.T., Nhiem N.X. (2023). Labdane- *type* diterpenoids and sesquiterpenes from *Curcuma aromatica* and their nitric oxide inhibitory activity in lipopolysaccharide-stimulated RAW264.7 macrophages. J. Asian Nat. Prod. Res..

[55] Park H.J., Kwon S.H., Yoo K.O., Sohn I.C., Lee K.T., Lee H.K. (2000). Sesquiterpenes from the leaves of *Ligularia fischeri* var. *spiciformis*. Planta Med..

[56] Descoins C., Bazzocchi I.L., Ravelo A.G. (2002). New Sesquiterpenes from *Euonymus europaeus* (*Celastraceae*). Chem. Pharm. Bull..

[57] Yang X.Y., Niu W.R., Li R.T., Cui X.M., Liu J.K. (2019). Two new sesquiterpenes from cultures of the higher fungus *Pholiota nameko*. Nat. Prod. Res..

[58] Shi Z.Z., Fang S.T., Miao F.P., Yin X.L., Ji N.Y. (2018). Trichocarotins A–H and trichocadinin A, nine sesquiterpenes from the marine-alga-epiphytic fungus *Trichoderma virens*. Bioorg. Chem..

[59] Rodriguez S., Kirby J., Denby C.M., Keasling J.D. (2014). Production and quantification of sesquiterpenes in Saccharomyces cerevisiae, including extraction, detection and quantification of terpene products and key related metabolites. Nat. Protoc..

[60] Song Y.P., Fang S.T., Miao F.P., Yin X.L., Ji N.Y. (2018). Diterpenes and Sesquiterpenes from the Marine Algicolous Fungus *Trichoderma harzianum* X-5. J. Nat. Prod..

[61] Torii M., Kato H., Hitora Y., Angkouw E.D., Mangindaan R.E.P., de Voogd N.J., Tsukamoto S. (2017). Lamellodysidines A and B, Sesquiterpenes Isolated from the Marine Sponge *Lamellodysidea herbacea*. J. Nat. Prod..

[62] Thebtaranonth C., Thebtaranonth Y., Wanauppathamkul S., Yuthavong Y. (1995). Antimalarial sesquiterpenes from tubers of *Cyperus rotundus*: Structure of 10,12-Peroxycalamenene, a sesquiterpene endoperoxide. Phytochemistry.

[63] Bartikova H., Hanusova V., Skalova L., Ambroz M., Bousova I. (2014). Antioxidant, pro-oxidant and other biological activities of sesquiterpenes. Curr. Top. Med. Chem..

[64] Nuermaimaiti M., Turak A., Yang Q., Tang B., Zang Y., Li J., Aisa H.A. (2021). Sesquiterpenes from *Artemisia Sieversiana* and their anti-inflammatory activities. Fitoterapia.

[65] Chi J., Li B.C., Dai W.F., Liu L., Zhang M. (2016). Highly oxidized sesquiterpenes from *Artemisia austro-yunnanensis*. Fitoterapia.

[66] Liang Y., Xu W., Liu C., Zhou D., Liu X., Qin Y., Cao F., Li J., Yang R., Qin J. (2019). Eremophilane sesquiterpenes from the endophytic fungus *Xylaria* sp. GDG-102. Nat. Prod. Res..

[67] Hansson D., Menkis A., Himmelstrand K., Thelander M., Olson Å., Stenlid J., Karlsson M., Broberg A. (2012). Sesquiterpenes from the conifer root rot pathogen *Heterobasidion occidentale*. Phytochemistry.

[68] Lim P.C., Ali Z., Khan I.A., Khan S.I., Kassim N.K., Awang K., Shaari K., Ismail A. (2022). Cytotoxic constituent of *Melicope latifolia* (DC.) T. G. Hartley. Nat. Prod. Res..

[69] Wu Z.l., Li J.Y., Sun Z.S., Yang Y.X., Xu X.K., Li H.L., Zhang W.D. (2022). Vlasouliodes A-D, four new C30 dimeric sesquiterpenes exhibiting potential inhibition of MCF-7 cells from *Vladimiria souliei*. Fitoterapia.

[70] Li C.S., Liu L.T., Yang L., Li J., Dong X. (2022). Chemistry and Bioactivity of Marine-Derived Bisabolane Sesquiterpenoids: A Review. Front. Chem..

[71] Hu S., Ma Y.L., Guo J.M., Wen Q., Yan G., Yang S., Fu Y.H., Liu Y.P. (2020). Bisabolane sesquiterpenes from Clausena sanki with their potential anti-inflammatory activities. Nat. Prod. Res..

[72] Shu H.Z., Peng C., Bu L., Guo L., Liu F., Xiong L. (2021). Bisabolane-type sesquiterpenoids: Structural diversity and biological activity. Phytochemistry.

[73] Liang X.T., Yu D.Q., Wu W.L., Deng H.C. (1979). The structure of yingzhaosu A. Acta Chim. Sin..

[74] Liang X.T., Yu D.Q., Pan W.D. (1979). The structure of yingzhaosu B. Acta Chim. Sin..

[75] Zhang L., Zhou W.S., Xu X. (1988). A new sesquiterpene peroxide (yingzhaosu C) and sesquiterpenol (yingzhaosu D) from *Artabotrys unciatus* (L.) Meer.. J. Chem. Soc. Chem. Commun..

[76] Xi F.M., Liu Y.B., Qu J., Li Y., Tang Z.H., Li L., Li Y.H., Chen X.G., Ma S.G., Yu S.S. (2017). Bioactive sesquiterpenoids from the roots of *Artabotrys hexapetalus*. Tetrahedron.

[77] Wang T.W. (2016). Study on Structures and Antitumor Activities of Chemical Constituents from *Artabotrys pilosus*. Master’s Thesis.

[78] Gu C., Yin A.P., Yuan H.Y., Yang K., Luo J., Zhan Y.J., Yang C.R., Zuo D.M., Li H.Z., Xu M. (2019). New anti-HBV norbisabolane sesquiterpenes from *Phyllantus acidus*. Fitoterapia.

[79] Wu S.L., Liu Y.P., Chen G.Y., Han C.R., Song X.P., Zhong X., Fu Y.H. (2017). Sesquiterpenes from *Artabotrys hongkongensis*. China J. Chin. Mater. Med..

[80] Jang H.J., Lee S., Lee S.J., Lim H.J., Jung K., Kim Y.H., Lee S.W., Rho M.C. (2017). Anti-inflammatory Activity of Eudesmane-Type Sesquiterpenoids from *Salvia plebeia*. J. Nat. Prod..

[81] Li W., Cai C.H., Guo Z.K., Wang H., Zuo W.J., Dong W.H., Mei W.L., Dai H.F. (2015). Five new eudesmane-type sesquiterpenoids from Chinese agarwood induced by artificial holing. Fitoterapia.

[82] Alarif W.M., Al-Footy K.O., Zubair M.S., Halid Ph M., Ghandourah M.A., Basaif S.A., Al-Lihaibi S.S., Ayyad S.E.N., Badria F.A. (2016). The role of new eudesmane-type sesquiterpenoid and known eudesmane derivatives from the red alga *Laurencia obtusa* as potential antifungal–antitumour agents. Nat. Prod. Res..

[83] Zhang J., Wang Y., Zhu R., Li Y., Li Y., Qiao Y., Zhou J., Lou H. (2018). Cyperane and eudesmane-type sesquiterpenoids from Chinese liverwort and their anti-diabetic nephropathy potential. RSC Adv..

[84] Ma L.F., Xu H., Wang J.D., Tong X.M., Zhan Z.J., Ying Y.M., Wang J.W., Zhang H., Shan W.G. (2018). Three new eudesmane sesquiterpenoids and a new dimer from the aerial part of *Salvia plebeia* R. Br.. Phytochem. Lett..

[85] Wen Q., Liu Y.P., Yan G., Yang S., Hu S., Hua J., Yin W.Q., Chen G.Y., Fu Y.H. (2020). Bioactive Eudesmane sesquiterpenes from *Artabotrys hongkongensis* Hance. Nat. Prod. Res..

[86] Tang J.Y. (2018). Study on the Chemical Constituents and Antitumor Activities of *Artabotrys hainanensis* R.E.Fries. Master’s Thesis.

[87] Li Y., Liu J., Wu Y., Li Y., Guo F. (2022). Guaiane-type sesquiterpenes from *Curcuma wenyujin*. Phytochemistry.

[88] Ma G.H., Chen K.X., Zhang L.Q., Li Y.M. (2019). Advance in biological activities of natural guaiane-type sesquiterpenes. Med. Chem. Res..

[89] Fleischer T.C., Waigh R.D., Waterman P.G. (1997). Pogostol O-methyl ether and artabotrol: Two novel sesquiterpenes from the stem bark of *Artabotrys stenopetalus*. J. Nat. Prod..

[90] Achenbach H., Schwinn A. (1995). Aporphinoid alkaloids and terpenoids from *Piptostigma fugax*. Phytochemistry.

[91] Chen G.Y., Zhu G.Y., Han C.R., Li Q.Y., Fang H.X., Bi H.P. (2005). Studies on sesquiterpenoids from the flowers of *Artabotrys hainanensis*. Chin. Tradit. Herb. Drugs.

[92] Peng G.P., Tian G., Huang X.F., Lou F.C. (2003). Guaiane-type sesquiterpenoids from *Alisma orientalis*. Phytochemistry.

[93] Ai H.L., Lv X., Ye K., Wang M.X., Huang R., Shi B.B., Li Z.H. (2022). Four New Highly Oxygenated Eremophilane Sesquiterpenes from an Endophytic Fungus *Boeremia exigua* Isolated from *Fritillaria hupehensis*. J. Fungi.

[94] Niu S., Liu D., Shao Z., Proksch P., Lin W. (2018). Eremophilane-type sesquiterpenoids in a deep-sea fungus *Eutypella* sp. activated by chemical epigenetic manipulation. Tetrahedron.

[95] Zhang W., Meng Q., Wu J., Cheng W., Liu D., Huang J., Fan A., Xu J., Lin W. (2022). Acorane sesquiterpenes from the deep-sea derived *Penicillium bilaiae* fungus with anti-neuroinflammatory effects. Front. Chem..

[96] Yong J.Y., Li M., Li W.R., Gao R.M., Su G.Z., Wang H.Q., Yang J., Li L., Li Y.H., Scott P. (2023). Seco-Sesquiterpenes and acorane-type sesquiterpenes with antiviral activity from the twigs and leaves of *Illicium henryi* Diels. Bioorg. Chem..

[97] Guo R., Ren Q., Tang Y.X., Zhao F., Lin B., Huang X.X., Song S.J. (2020). Sesquiterpenoids from the roots of *Daphne genkwa* Siebold et Zucc. with potential anti-inflammatory activity. Phytochemistry.

[98] Zhang M., Zhao J.L., Liu J.M., Chen R.D., Xie K.B., Chen D.W., Feng K.P., Zhang D., Dai J.G. (2017). Neural anti-inflammatory sesquiterpenoids from the endophytic fungus *Trichoderma* sp. Xy24. J. Asian Nat. Prod. Res..

[99] Jiang L., Wen Y., Peng Y., Chen T., Chen J., Yang J., Gong T., Zhu P. (2021). Advances in biosynthesis of cadinane sesquiterpenes. Chin. J. Biotechnol..

[100] Zhou C.X., Zhang L.S., Chen F.F., Wu H.S., Mo J.X., Gan L.S. (2017). Terpenoids from *Curcuma wenyujin* increased glucose consumption on HepG2 cells. Fitoterapia.

[101] Shang Z.C., Han C., Xu J.L., Liu R.H., Yin Y., Wang X.B., Yang M.H., Kong L.Y. (2019). Twelve formyl phloroglucinol meroterpenoids from the leaves of *Eucalyptus robusta*. Phytochemistry.

[102] Qin D.P., Pan D.B., Xiao W., Li H.B., Yang B., Yao X.J., Dai Y., Yu Y., Yao X.S. (2018). Dimeric Cadinane Sesquiterpenoid Derivatives from *Artemisia annua*. Org. Lett..

[103] Paddon C.J., Westfall P.J., Pitera D.J., Benjamin K., Fisher K., McPhee D., Leavell M.D., Tai A., Main A., Eng D. (2013). High-level semi-synthetic production of the potent antimalarial artemisinin. Nature.

[104] Wang L.X., Jiang X.J., Li X.M., Mao M.F., Wei G.Z., Wang F. (2019). Aristolane-type Sesquiterpenoids from *Nardostachys chinensis* and Revised Structure of *Aristolanhydride*. Nat. Prod. Bioprospect..

[105] Durán-Peña M.J., Botubol Ares J.M., Hanson J.R., Collado I.G., Hernández-Galán R. (2015). Biological activity of natural sesquiterpenoids containing a gem-dimethylcyclopropane unit. Nat. Prod. Rep..

[106] Chen Y.P., Ying S.S., Zheng H.H., Liu Y.T., Wang Z.P., Zhang H., Deng X., Wu Y.J., Gao X.M., Li T.X. (2017). Novel serotonin transporter regulators: Natural aristolane- and nardosinane- types of sesquiterpenoids from *Nardostachys chinensis* Batal. Sci. Rep..

[107] Lan Y.H., Wang H.Y., Wu C.C., Chen S.L., Chang C.L., Chang F.R., Wu Y.C. (2007). New Constituents from Stems of *Artabotrys uncinatus*. Chem. Pharm. Bull..

[108] Shen S.M., Yang Q., Zang Y., Li J., Liu X., Guo Y.W. (2022). Anti-inflammatory aromadendrane-and cadinane-type sesquiterpenoids from the South China Sea sponge *Acanthella cavernosa*. Beilstein J. Org. Chem..

[109] Zhang C.Y., Zhang J.Z., Li Y.L., Xu Z.J., Qiao Y.N., Yuan S.Z., Tang Y.J., Lou H.X. (2024). Heterodimers of Aromadendrane Sesquiterpenoid with Benzoquinone from the Chinese Liverwort *Mylia nuda*. J. Nat. Prod..

[110] Mahidol C., Chimnoi N., Chokchaichamnankit D., Techasakul S. (2005). Identification of volatile constituents in *Artabotrys hexapetalus* flowers using simple headspace solvent-trapping technique in combination with gas chromatography-mass spectrometry and retention indices. Acta Hortic..

[111] Brabin B.J. (2014). Malaria’s contribution to World War One–the unexpected adversary. Malar. J..

[112] Liu Y., He Z.Q., Wang D., Hu Y.B., Qian D., Yang C.Y., Zhou R.M., Li S.H., Lu D.L., Zhang H.W. (2022). One Health approach to improve the malaria elimination programme in Henan Province. Adv. Parasitol..

[113] Garrido-Cardenas J.A., González-Cerón L., Manzano-Agugliaro F., Mesa-Valle C. (2019). *Plasmodium* Genomics: An approach for learning about and ending human malaria. Parasitol. Res..

[114] Barber B.E., Grigg M.J., Cooper D.J., van Schalkwyk D.A., William T., Rajahram G.S., Anstey N.M. (2021). Clinical management of Plasmodium knowlesi malaria. Adv. Parasitol..

[115] Fuehrer H.P., Campino S., Sutherland C.J. (2022). The primate malaria parasites *Plasmodium malariae*, *Plasmodium brasilianum* and *Plasmodium ovale* spp.: Genomic insights into distribution, dispersal and host transitions. Malar. J..

[116] Cohen S. (1979). Immunity to malaria. Proc. R. Soc. Lond. B Biol. Sci..

[117] Walker I.S., Rogerson S.J. (2023). Pathogenicity and virulence of malaria: Sticky problems and tricky solutions. Virulence.

[118] White N.J. (2008). The role of anti-malarial drugs in eliminating malaria. Malar. J..

[119] Kong L.Y., Tan R.X. (2015). Artemisinin, a miracle of traditional Chinese medicine. Nat. Prod. Rep..

[120] Bailly C., Hénichart J.P. (2022). Advocacy for the Medicinal Plant *Artabotrys hexapetalus* (Yingzhao) and Antimalarial Yingzhaosu Endoperoxides. Molecules.

[121] Liu S., Wei C., Liu T., Ma S.G., Chen C., Lin H., Zhang L., Wang H., Zhang C.J., Yu S.S. (2022). A heme-activatable probe and its application in the high-throughput screening of *Plasmodium falciparum* ring-stage inhibitors. Signal Transduct. Tar..

[122] Boukouvalas J., Pouliot R., Fréchette Y. (1995). Concise synthesis of yingzhaosu C and epi-yingzhaosu C by peroxyl radical cyclization. Assignment of relative configuration. Tetrahedron Lett..

[123] Xu X., Xie X. (2010). Total synthesis of Yingzhaosu B and its three diastereoisomers. Chin. J. Chem..

[124] Xu X.X., Hu Q.S. (1992). Synthesis of the diastereoisomeric Yingzhaosu D. Chin. J. Chem..

[125] Zhang G.F., Liu X., Zhang S., Pan B., Liu M.L. (2018). Ciprofloxacin derivatives and their antibacterial activities. Eur. J. Med. Chem..

[126] Li Z., Zhao L., Bian Y., Li Y., Qu J., Song F. (2022). The Antibacterial Activity of Quinazoline and Quinazolinone Hybrids. Curr. Top. Med. Chem..

[127] Kaur R., Rani P., Atanasov A.G., Alzahrani Q., Gupta R., Kapoor B., Gulati M., Chawla P. (2022). Discovery and Development of Antibacterial Agents: Fortuitous and Designed. Mini Rev. Med. Chem..

[128] D’Agostino I., Ardino C., Poli G., Sannio F., Lucidi M., Poggialini F., Visaggio D., Rango E., Filippi S., Petricci E. (2022). Antibacterial alkylguanidino ureas: Molecular simplification approach, searching for membrane-based MoA. Eur. J. Med. Chem..

[129] Paulin S., Alm R.A., Beyer P. (2020). A novel pre-clinical antibacterial pipeline database. PLoS ONE.

[130] Vila J., Moreno-Morales J., Ballesté-Delpierre C. (2020). Current landscape in the discovery of novel antibacterial agents. Clin. Microbiol. Infect..

[131] Cattoir V., Felden B. (2019). Future Antibacterial Strategies: From Basic Concepts to Clinical Challenges. J. Infect. Dis..

[132] Nyandoro S.S., Joseph C.C., Nkunya M.H.H., Hosea K.M.M. (2013). New antimicrobial, mosquito larvicidal and other metabolites from two *Artabotrys* species. Nat. Prod. Res..

[133] Tan M., Zhou L., Huang Y., Wang Y., Hao X., Wang J. (2008). Antimicrobial activity of globulol isolated from the fruits of Eucalyptus globulus Labill. Nat. Prod. Res..

[134] Chulalaksananukul W. (2014). Chemical composition and antibacterial activity of extracts from freshwater green algae, Cladophora glomerata Kützing and Microspora floccosa (Vaucher) Thuret. J. BioScience Biotechnol..

[135] Debela D.T., Muzazu S.G.Y., Heraro K.D., Ndalama M.T., Mesele B.W., Haile D.C., Kitui S.K., Manyazewal T. (2021). New approaches and procedures for cancer treatment: Current perspectives. SAGE Open Med..

[136] Liu X., Tian F., Zhang H.B., Pilarinou E., McLaughlin J.L. (1999). Biologically Active Blumenol A from the Leaves of *Annona Glabra*. Nat. Prod. Rep..

[137] Das M., Prakash S., Nayak C., Thangavel N., Singh S.K., Manisankar P., Devi K.P. (2018). Dihydroactinidiolide, a natural product against Aβ_25–35_ induced toxicity in Neuro2a cells: Synthesis, in silico and in vitro studies. Bioorg. Chem..

[138] Park K.R., Nam D., Yun H.M., Lee S.G., Jang H.J., Sethi G., Cho S.K., Ahn K.S. (2011). β-Caryophyllene oxide inhibits growth and induces apoptosis through the suppression of PI3K/AKT/mTOR/S6K1 pathways and ROS-mediated MAPKs activation. Cancer Lett..

[139] Martin G.S. (2003). Cell signaling and cancer. Cancer Cell.

[140] Castaneda C.A., Cortes-Funes H., Gomez H.L., Ciruelos E.M. (2010). The phosphatidyl inositol 3-kinase/AKT signaling pathway in breast cancer. Cancer Metastasis Rev..

[141] Sanjeewa K.K.A., Herath K.H.I.N.M., Yang H.W., Choi C.S., Jeon Y.J. (2021). Anti-Inflammatory Mechanisms of Fucoidans to Treat Inflammatory Diseases: A Review. Mar. Drugs.

[142] Hou C., Chen L., Yang L., Ji X. (2020). An insight into anti-inflammatory effects of natural polysaccharides. Int. J. Biol. Macromol..

[143] Rocha D.H.A., Pinto D.C.G.A., Silva A.M.S. (2022). Macroalgae Specialized Metabolites: Evidence for Their Anti-Inflammatory Health Benefits. Mar. Drugs.

[144] Dinarello C.A. (2010). Anti-inflammatory Agents: Present and Future. Cell.

[145] Kazemi S., Shirzad H., Rafieian-Kopaei M. (2018). Recent Findings in Molecular Basis of Inflammation and Anti-inflammatory Plants. Curr. Pharm. Des..

[146] Gautam R., Jachak S.M. (2009). Recent developments in anti-inflammatory natural products. Med. Res. Rev..

[147] Cruz-Martins N. (2022). Molecular Mechanisms of Anti-Inflammatory Phytochemicals. Int. J. Mol. Sci..

[148] Pekacar S., Bulut S., Özüpek B., Orhan D.D. (2021). Anti-Inflammatory and Analgesic Effects of Rosehip in Inflammatory Musculoskeletal Disorders and Its Active Molecules. Curr. Mol. Pharmacol..

[149] Elbandy M. (2023). Anti-Inflammatory Effects of Marine Bioactive Compounds and Their Potential as Functional Food Ingredients in the Prevention and Treatment of Neuroinflammatory Disorders. Molecules.

[150] Jang J.H., Lee T.J. (2023). Mechanisms of Phytochemicals in Anti-Inflammatory and Anti-Cancer. Int. J. Mol. Sci..

[151] Delgado G., del Socorro Olivares M., Chávez M.I., Ramírez-Apan T., Linares E., Bye R., Espinosa-García F.J. (2001). Antiinflammatory Constituents from *Heterotheca inuloides*. J. Nat. Prod..

[152] do Nascimento K.F., Moreira F.M.F., Alencar Santos J., Kassuya C.A.L., Croda J.H.R., Cardoso C.A.L., Vieira M.D.C., Góis Ruiz A.L.T., Ann Foglio M., de Carvalho J.E. (2018). Antioxidant, anti-inflammatory, antiproliferative and antimycobacterial activities of the essential oil of *Psidium guineense* Sw. and spathulenol. J. Ethnopharmacol..

[153] Shi D., Song X., Guo Y., Xu J., Liu Y., Zhang J., Cui C.A., Jin D.Q. (2017). Alismol, a Sesquiterpenoid Isolated from Vladimiria souliei, Suppresses Proinflammatory Mediators in Lipopolysaccharide-Stimulated Microglia. J. Mol. Neurosci..

[154] Kumari R., Sharma S.D., Kumar A., Ende Z., Mishina M., Wang Y., Falls Z., Samudrala R., Pohl J., Knight P.R. (2023). Antiviral Approaches against Influenza Virus. Clin. Microbiol. Rev..

[155] Gudima G., Kofiadi I., Shilovskiy I., Kudlay D., Khaitov M. (2023). Antiviral Therapy of COVID-19. Int. J. Mol. Sci..

[156] Beheshtirouy S., Khani E., Khiali S., Entezari-Maleki T. (2022). Investigational antiviral drugs for the treatment of COVID-19 patients. Arch. Virol..

[157] Chareonkla A., Pohmakotr M., Reutrakul V., Yoosook C., Kasisit J., Napaswad C., Tuchinda P. (2011). A new diarylheptanoid from the rhizomes of *Zingiber mekongense*. Fitoterapia.

[158] Yanda L., Tatsimo S.J.N., Tamokou J.D.D., Matsuete-Takongmo G., Meffo-Dongmo S.C., Meli Lannang A., Sewald N. (2022). Antibacterial and Antioxidant Activities of Isolated Compounds from *Prosopis africana* Leaves. Int. J. Anal. Chem..

[159] Chaudhary A.K., Ahmad S., Mazumder A. (2015). Isolation, structural elucidation and in vitro antioxidant activity of compounds from chloroform extract of *Cedrus deodara* (Roxb.) Loud. Nat. Prod. Res..

